# MULTICAUSENET temporal attention for multimodal emotion cause pair extraction

**DOI:** 10.1038/s41598-025-01221-w

**Published:** 2025-06-03

**Authors:** Ma Junchi, Hassan Nazeer Chaudhry, Farzana Kulsoom, Yang Guihua, Sajid Ullah Khan, Sujit Biswas, Zahid Ullah Khan, Faheem Khan

**Affiliations:** 1 School of Mechatronics Engineering, Daqing Normal University, Daqing, 163712 China; 2 Department of Electronics, Information and Bioengineering, Politecnico di Milano, Milan, Italy; 3 Department of Telecommunication Engineering, University of Engineering and Technology, Taxila, Pakistan; 4 Department of Information Systems, College of Computer Engineering and Sciences, Prince Sattam Bin Abdulaziz University, Alkharj, 16273 Saudi Arabia; 5 College of Information and Communication Engineering, Harbin Engineering University, Harbin, 150001 China; 6 Department of Computer Engineering, Gachon University, Seongnam-si, 13120 South Korea; 7 Computer Science Department, City St. George’s University of London, London, United Kingdom

**Keywords:** Emotion–cause pair extraction, Multimodal emotion recognition, Graph attention networks (GATs), Vision transformers (ViTs), Transformers and attention mechanisms, Feature fusion, Multimodal graphs, Self and cross attention, Emotion triggers, Health care, Medical research, Signs and symptoms

## Abstract

In the realm of emotion recognition, understanding the intricate relationships between emotions and their underlying causes remains a significant challenge. This paper presents MultiCauseNet, a novel framework designed to effectively extract emotion-cause pairs by leveraging multimodal data, including text, audio, and video. The proposed approach integrates advanced multimodal feature extraction techniques with attention mechanisms to enhance the understanding of emotional contexts. The key text, audio, and video features are extracted using BERT, Wav2Vec, and Vision transformers (ViTs), which are then employed to construct a comprehensive multimodal graph. The graph encodes the relationships between emotions and potential causes, and Graph Attention Networks (GATs) are used to weigh and prioritize relevant features across the modalities. To further improve performance, Transformers are employed to model intra-modal and inter-modal dependencies through self-attention and cross-attention mechanisms. This enables a more robust multimodal information fusion, capturing the global context of emotional interactions. This dynamic attention mechanism enables MultiCauseNet to capture complex interactions between emotional triggers and causes, improving extraction accuracy. Experiments on emotion benchmark datasets, including IEMOCAP and MELD achieved a WFI score of 73.02 and 53.67 respectively. The results for cause pair analysis are evaluated on ECF and ConvECPE with a Cause recognition F1 score of 65.12 and 84.51, and a Pair extraction F1 score of 55.12 and 51.34.

## Introduction

**Fig. 1:**
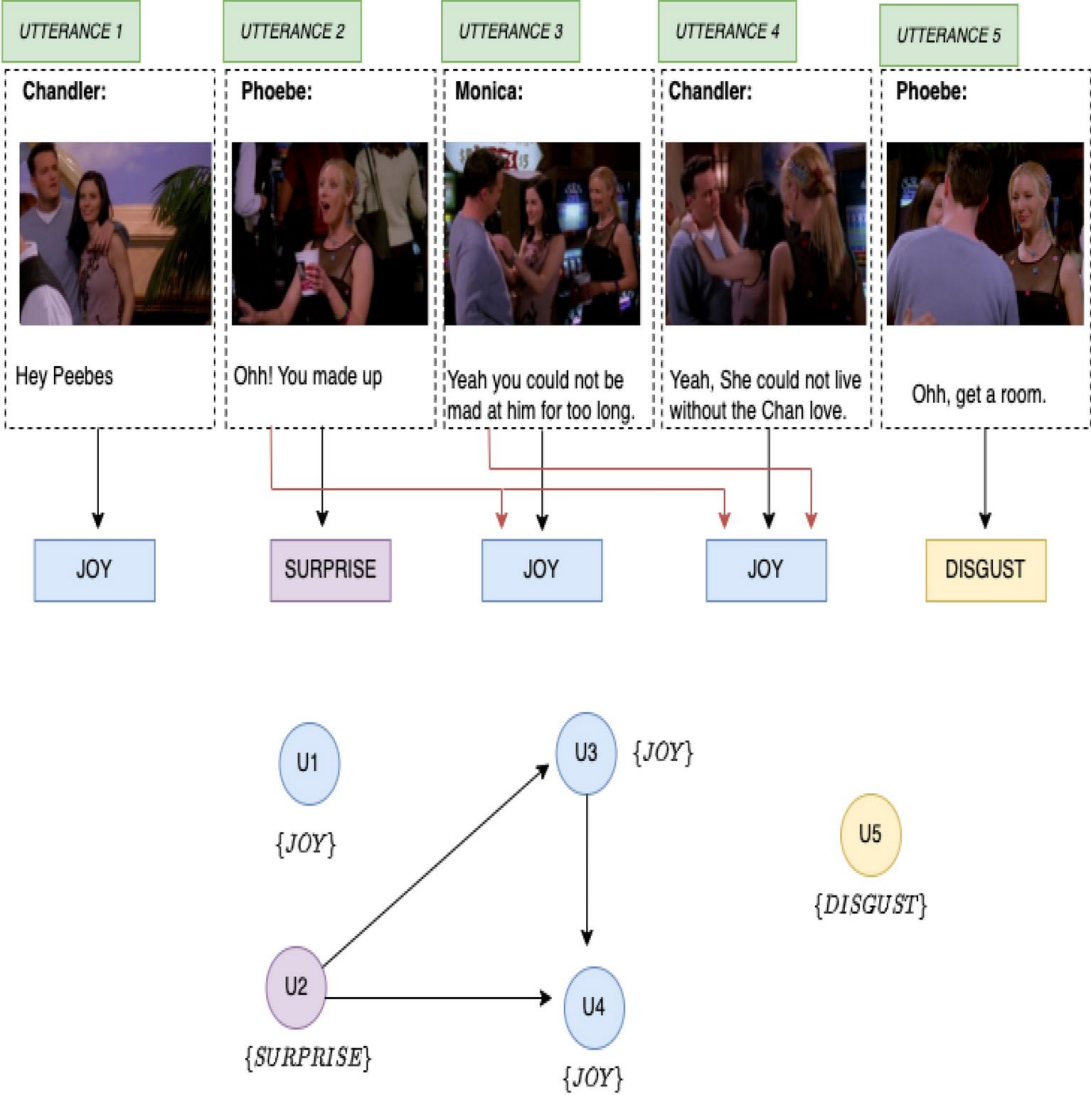
Example of cause analysis.

In recent years, understanding human emotions has gained increasing attention across multiple domains. Emotion recognition has evolved from merely identifying feelings to analyzing their complexities, intricacies, and causal relationships with specific triggers^1^. The ability to link emotions with their corresponding causes-termed emotion–cause pair extraction-has substantial implications in various applications, such as sentiment analysis, social media monitoring, and mental health assessment. To understand emotion–cause Pair Extraction consider a scenario in which a user posts a message on social media expressing sadness after watching a particular film. An emotion–cause pair extraction system could identify the emotion (“sadness”) and link it to the cause (“watching a film”). This capability not only aids in understanding the user’s emotional state but also the reasons behind it. Another example is a user who shares their experience of feeling overwhelmed during a job interview. An emotion–cause extraction system would capture the emotion (“overwhelmed”) and the cause (“job interview”). As a third example, we will use a visual description. Figure 1 illustrates a sequence of events involving five utterances, each represented by an image at the top. These utterances correspond to emotional responses that evolve throughout the interaction. Utterance 1 shows a moment of joy, with the characters appearing happy. In Utterance 2, the emotional tone shifts to surprise, as one of the characters reacts unexpectedly. The emotion returns to joy in Utterances 3 and 4, with the characters exhibiting positive feelings. However, in Utterance 5, there is a shift to disgust, as one of the characters expresses a negative reaction. Below the emotional labels for each utterance, a chain of causal relationships between the utterances is shown. These utterances are labelled U1 through U5 and connected by arrows, indicating the progression of emotions. Utterance 1, which is linked to joy, causes the surprise seen in Utterance 2. This surprise, in turn, leads to a joyful response in Utterance 3. The joy continues into Utterance 4, showing a flow of positive emotions.

However, this emotional flow is broken in Utterance 5, where the emotion shifts to disgust. The arrows connecting the emotions, such as joy, surprise, and disgust, highlight the way one emotional state triggers the next. For example, the surprise experienced in Utterance 2 leads to a joyful reaction in Utterances 3 and 4, before finally transitioning to disgust in Utterance 5. This causal structure represents the dynamic nature of emotional interactions in a conversation, showing how emotions influence one another as the dialogue unfolds. The overall figure demonstrates how different utterances cause shifts in emotional states, contributing to the unfolding emotional narrative.

The emotion extraction could be done using a single modality such as image, text and video, or a combination of these or more modalities^2^. The distinction between single and multiple modalities in emotion recognition is critical. Single-modality approaches typically focus on one form of data, such as text or audio, to identify emotions. While these methods can yield useful insights, they often fall short of capturing the full complexity of human emotions. For example, relying solely on textual data may miss vital emotional cues present in tone or facial expressions^3^. Conversely, multiple modalities offer a more holistic view of emotional expressions. By integrating data from various sources, researchers can construct a comprehensive understanding of emotions. For instance, a study by^4^ successfully combined audio, visual, and textual features to enhance emotion recognition performance, demonstrating the efficacy of multimodal approaches. However, the integration of multiple modalities poses its challenges, including the need for effective feature extraction, alignment of temporal sequences, the handling of missing or noisy data across different modalities and having images of low quality^5,6^. These challenges necessitate advanced methodologies capable of addressing the complexities inherent in multimodal data. Human emotions are inherently multimodal, manifested not just through spoken or written words but also through tone, body language, facial expressions, and contextual cues from video content. As a result, leveraging multimodal data is essential for capturing the richness of emotional expressions.

Traditional approaches often focus on isolated modalities, neglecting the temporal relationships that interconnect these inputs. Consequently, there is a significant gap in the literature regarding integrating multiple modalities for effective emotion–cause pair extraction. Despite advancements in emotion recognition, several challenges hinder effective emotion–cause pair extraction. Firstly, the inherent subjectivity of emotions complicates the development of robust models capable of generalizing across diverse contexts and individual differences. Emotions are often nuanced and context-dependent, making it challenging to establish clear causal relationships. For instance, what elicits joy in one individual may evoke sadness in another, depending on their unique experiences and perspectives. Secondly, existing methodologies frequently rely on a single modality, which limits their effectiveness. For instance, textual analysis alone may not capture the full spectrum of emotional expression, as tone and context are equally significant. This limitation is particularly pronounced in dynamic settings, where real-time interactions necessitate a comprehensive understanding of multiple modalities. A singular focus on one modality can lead to incomplete analyses, as it ignores the potential contributions of other forms of data. Thirdly, the temporal aspect of emotions further complicates the extraction process. Emotions are not static; they evolve, influenced by preceding events and ongoing stimuli. As such, capturing the temporal relationships between different modalities is essential for accurately linking emotions with their causes. Fourthly, image quality is very important for correct extraction of cause and emotions^7^. There is rich literature on image quality inspection which could be employed to assess the quality of the image before cause or emotion could be determined^8–10^.

Traditional machine-learning approaches often fail to account for these temporal dynamics, leading to incomplete or misleading conclusions. The motivation behind this research stems from the understanding that emotions are complex constructs influenced by various factors, including situational context and individual differences. For instance, a person’s emotional reaction to a film scene may depend on both the auditory cues (e.g., background music) and visual elements (e.g., facial expressions of characters). Furthermore, the context in which these cues are presented plays a critical role in shaping emotional responses. Therefore, an integrated approach that considers the interplay between text, audio, and video is crucial for accurately identifying emotions and their causes. This integration can provide a more nuanced understanding of emotional experiences, facilitating insights that are often lost in unidimensional analyses. Moreover, the significance of emotion–cause pair extraction extends beyond academic interest; it holds practical implications for industries such as marketing, where understanding customer emotions can lead to more effective strategies, and healthcare, where emotional assessments can enhance patient care. For example, in mental health assessments, accurately identifying emotions can inform therapeutic approaches, leading to improved patient outcomes. By elucidating the triggers of emotions, we can foster a deeper understanding of human behaviour and improve decision-making processes in various fields. Several studies have focused on emotion recognition, utilizing various methodologies and datasets. Early works primarily relied on traditional machine learning techniques, such as support vector machines (SVM) and hidden Markov models (HMM), for emotion classification based on textual data. For example,^11^ explored the use of lexical features in the text to identify emotions, while^12^ demonstrated the effectiveness of HMM for recognizing emotions in speech. With the advent of deep learning, significant advancements have been made in this field. Convolutional Neural Networks (CNNs) and Recurrent Neural Networks (RNNs) have been widely adopted for emotion recognition tasks.^13^ proposed a deep learning framework combining CNNs and LSTMs to extract features from both text and audio for emotion classification.

Recent advancements in transformer architectures have also significantly influenced emotion recognition tasks. Models such as BERT and its variations have demonstrated state-of-the-art performance in various natural language processing tasks, including emotion detection^14^. Furthermore, the integration of transformers with audio and visual modalities has shown promise in capturing the contextual relationships essential for emotion–cause pair extraction.

In addressing these challenges, this paper proposes a novel technique that leverages attention mechanisms and graph-based representations to enhance multimodal emotion–cause pair extraction. The proposed system integrates text, audio, and video inputs to construct a comprehensive graph representation that captures emotions’ relationships and potential triggers. The process begins with multimodal feature extraction, where contextual embeddings for text, audio, and video are generated using state-of-the-art models such as BERT, Wav2Vec, and Vision Transformers (ViT). BERT^14^ has been extensively used for text representation, capturing contextual information effectively. Wav2Vec^15^ excels in audio feature extraction by leveraging self-supervised learning to model acoustic representations. Similarly, Vision Transformers^16^ have shown promising results in extracting visual features from video data. These embeddings are then employed to construct a multimodal graph, where vertices represent key features across modalities, and edges encode the relationships between emotions and their potential causes. The graph structure represents complex interconnections between different emotional triggers, enhancing the model’s ability to capture the nuanced relationships inherent in human emotions. Subsequently, we employ Graph Attention Networks (GAT) to facilitate the emotion–cause pairing process. GATs leverage attention mechanisms to assign different weights to the vertices in the graph, enabling the model to focus on the most relevant features while accounting for the temporal relationships inherent in multimodal data. This dynamic attention mechanism enhances the model’s ability to adaptively learn from the data, leading to improved performance in emotion–cause pair extraction.

The resultant framework not only enhances the accuracy of emotion–cause pair extraction but also provides valuable insights into the contextual interplay between emotions and their triggers. By effectively capturing the relationships among different modalities, our proposed technique aims to bridge the existing gap in the literature and advance the field of emotion recognition. In conclusion, this research contributes to the growing body of knowledge in the field of emotion recognition by introducing a robust and integrative approach to emotion–cause pair extraction. Through the application of multimodal inputs and advanced attention mechanisms, we aim to advance the understanding of emotions and their complexities in real-world scenarios. By elucidating the triggers of emotions, we hope to pave the way for future research and applications in this vital area of study. Furthermore, our findings underscore the necessity of developing more sophisticated models that can handle the intricacies of human emotions in dynamic environments. This research lays the groundwork for further exploration in emotion recognition and highlights the potential for transformative applications in various sectors, including mental health, marketing, and human-computer interaction, where understanding emotional nuances is essential for fostering positive experiences and outcomes. The objectives of this work are to develop a novel multimodal framework for emotion–cause pair extraction by integrating text, audio, and video features using advanced models such as BERT, Wav2Vec, and ViT. The proposed approach aims to construct a multimodal graph representation that effectively models the relationships between emotions and their causes. By introducing a temporal attention mechanism and leveraging GATs, this research seeks to align multimodal features over time, enhance dynamic feature linking, and capture complex dependencies between emotional states and triggers. Additionally, the study aims to demonstrate the superiority of the proposed method through a comprehensive evaluation of benchmark datasets, contributing to the field by setting a new benchmark for emotion–cause pair extraction.

To summarize, our key contributions are:

- We propose a novel multimodal framework for emotion–cause pair extraction, integrating text, audio, and video features using BERT, Wav2Vec, and ViT. A multimodal graph representation models emotion–cause relationships, with vertices for modality-specific features and edges capturing their interactions.
- We introduce a temporal attention mechanism that aligns multimodal features over time, enabling the model to account for the evolving nature of emotions and their causes across different modalities.
- We integrate GATs for dynamic attention-weighted feature linking and combine Transformers for global context with GATs for local relational modelling, enabling enhanced intra-modal and inter-modal fusion. This unified architecture effectively captures complex dependencies between emotions and their causes.
- We provide a comprehensive analysis of the temporal and contextual dynamics of emotions, showing how the model captures emotion evolution and the intricate dependencies between emotional states and their causes, setting a new benchmark for future research in this domain.
- Evaluated on IEMOCAP and MELD, our framework outperforms existing methods by leveraging multimodal strengths. It also captures temporal and contextual emotion dynamics, setting a new benchmark for emotion–cause extraction.

Section “Related work” presents existing literature and state of the art; Section “Proposed Technique” describes the proposed technique. Section “Results and performance evaluation”, presents the experimental setup and discusses the results. Finally, Section “Conclusions” concludes.

## Related work

This section presents the related work to multimodal emotion recognition and cause pair analysis. Section “Multimodal emotion recognition” provides a brief introduction to multimodal emotion recognition. Section “Emotion cause pair extraction” explains research work related to cause pair analysis. The last Section “Benchmark state of art” provides a short introduction to research work used as a bench mark in the results section.

**Table 1:** Benchmarking MultiCauseNet against baseline methods.

Method (year)	Deep learning technique	Dataset	Notable aspects
DialogueGCN (2019)^17^	Graph Convolutional Network (GCN)	IEMOCAP	Models interrelations among dialogue turns
DialogueRNN (2019)^18^	Recurrent Neural Network (RNN)	IEMOCAP, MELD	Captures sequential dynamics of dialogue
MMGCN (2019)^19^	Multimodal GCN	IEMOCAP	Enhances recognition for Sadness and Excitement
IterativeERC (2020)^20^	Iterative Method	IEMOCAP	Refines predictions through multiple iterations
QMNN (2021)^21^	Quantum-Inspired Techniques	Various	Integrates techniques across modalities
MM-DFN (2022)^22^	Deep Fusion Network	IEMOCAP	Addresses complex emotional expressions
MVN (2022)^23^	Multi-View Approach	Various	Extracts diverse emotional signals
UniMSE (2022)^24^	Self-Supervised Learning	Various	Unified multimodal strategy
EmoCaps (2022)^2^	Various	Various	Detects nuanced emotional expressions
GA2MIF (2023)^25^	Facial and Contextual Info	Various	Enhances emotion recognition
MALN (2023)^26^	Multimodal Learning Network	Various	Excels in recognizing multiple emotions
MultiEMO (2023)^27^	Advanced Methodology	Various	Excels in detecting Sad emotions

### Multimodal emotion recognition

MERC can be categorized into three primary groups: multimodal fusion, context-aware models, and studies integrating external knowledge. The first group focuses on fusion representations. Some works, such as Hu et al.^28,29^ and Joshi et al.^30^, employ graph neural networks to model the inter- and intra-dependencies of utterance information. Additionally, other studies propose cross-attention Transformers^31^ to capture cross-modality interactions. In addressing context incorporation, Sun et al.^32^, Li et al.^33^, and Ghosal et al.^34^ construct graph structures to represent contextual information and model inter-utterance dependencies. Furthermore, Mao et al.^35^ introduce the concept of emotion dynamics to effectively capture context. The final group includes advanced MERC studies that integrate external knowledge. These studies utilize techniques such as transfer learning^33,36^, commonsense knowledge^37^, multi-task learning^38^, and external information^39^ to provide auxiliary information, enhancing the model’s understanding of conversations.

### Emotion cause pair extraction

With the growing trend of extending various NLP tasks to the multimodal domain^28,40–43^, Wang et al.^44^ introduced the concept of MECPE and created the Emotion–Cause-in-Friends (ECF) dataset, which is derived from the MELD dataset^4^. In addition, Li et al.^45^ developed a multimodal dataset for English conversational emotion–cause pair extraction, leveraging the IEMOCAP dataset^46^. The primary objective of MECPE is to determine the cause of utterances corresponding to a given emotion utterance, thereby generating pairs of utterances. Despite the recent emergence of MECPE as a research area, there is a limited number of baseline methods available. In earlier works, Wang et al.^44^ and Li et al.^45^ established baseline approaches by integrating multimodal features to address the MECPE task. Although these studies broadened the scope of emotion–cause pair extraction to a multimodal context, they primarily adapted existing baseline methods designed for text-based emotion–cause extraction, neglecting the critical roles of inter-utterance context and multimodal fusion in effectively understanding emotional causation.

### Benchmark state of art

Table 1 provides an overview of various baseline methods employed in emotion recognition, particularly contrasting these with the proposed MultiCauseNet model. Each method showcases distinct deep learning techniques, datasets utilized, and notable aspects that contribute to the field of emotion recognition. The **DialogueGCN** method employs GCNs to capture the interrelations among dialogue turns. By leveraging the structural properties of graphs, this approach enhances the contextual understanding of emotional exchanges within conversations. Utilizing the IEMOCAP dataset, this method is effective in discerning the nuances of emotional expression by mapping relationships between various dialogue participants. Similarly, **DialogueRNN** leverages RNNs to encapsulate the sequential dynamics inherent in dialogue. Its implementation across both the IEMOCAP and MELD datasets signifies its robustness in managing diverse dialogue structures. This capability is crucial for tracking emotional transitions and reactions throughout conversations, thereby providing a richer analysis of emotional development over time.

In addition, **MMGCN** adopts a Multimodal GCN to enhance recognition capabilities for emotions such as sadness and excitement. By integrating multimodal features, this method addresses the complexity of emotional detection, demonstrating improved performance across various emotional categories through the incorporation of diverse input types, including audio and visual data. The **IterativeERC** method introduces an iterative refinement process, allowing for continuous improvement of predictions through feedback mechanisms. This approach highlights the significance of adaptive learning in dynamic dialogue contexts, enhancing the model’s understanding and responsiveness to emotional cues effectively. The **QMNN** showcases an innovative blend of quantum computing and machine learning, utilizing quantum-inspired techniques to bolster multimodal integration. By effectively merging different modalities, this method expands the scope of emotion recognition, suggesting that advanced computational frameworks can significantly enhance the efficacy of emotion detection systems. On the other hand, employing a Deep Fusion Network, **MM-DFN** addresses the challenges associated with recognizing complex emotional expressions. This method’s focus on the fusion of multimodal information emphasizes the necessity of combining various data sources to attain a comprehensive understanding of emotional states, which are often multifaceted.

Further, the **MVN** method implements a multi-view approach to extract a range of emotional signals. By analyzing data from diverse perspectives, this technique enhances the model’s ability to recognize emotions across varying contexts, indicating the benefits of adopting a holistic view in emotional analysis. Utilizing self-supervised learning, **UniMSE** unifies multimodal strategies, minimizing the dependence on extensive labelled datasets. This approach is vital for scaling emotion recognition systems, as it facilitates learning from unstructured data while maintaining high performance and accuracy. Moreover, the **EmoCaps** method is dedicated to detecting nuanced emotional expressions, utilizing various techniques across multiple datasets. This focus on subtle emotional cues is crucial, as they often convey significant information that might be overlooked by conventional emotion recognition systems. The **GA2MIF** method enhances emotion recognition by merging facial expressions with contextual information. This dual-focus approach fosters a comprehensive understanding of emotional cues, which is essential for applications demanding high accuracy in emotional detection, such as in social interactions and mental health monitoring. The **MALN** excels in recognizing multiple emotions simultaneously through a Multimodal Learning Network. This capability to process and integrate diverse information sources effectively positions this method as a significant advancement in the field, catering to the complexities of human emotional expression. Finally, **MultiEMO** emphasizes the detection of sadness, employing advanced methodologies that improve recognition accuracy within multimodal frameworks. This targeted approach underscores the growing necessity for emotion recognition systems to deeply understand specific emotional states, particularly in sensitive applications like mental health monitoring.

## Proposed technique

This section describes the proposed technique for multimodal emotion–cause pair extraction, which leverages various state-of-the-art models to process and integrate information from different modalities. The approach is structured into multiple stages, starting from feature extraction to final emotion–cause pair identification, and each stage plays a critical role in achieving robust performance across modalities. The first section “Multimodal feature extraction”, discusses the extraction of features from three modalities: text, audio, and video. Each modality’s independent features are then aligned and integrated for subsequent stages of analysis. Following feature extraction, the next stage, detailed in Section “Feature fusion using transformers”, focuses on the fusion of multimodal features using attention mechanisms. The fusion process combines intra-modal and inter-modal dependencies, enabling the model to represent complex relationships across text, audio, and video modalities. The self-attention mechanism captures intra-modal dependencies, while cross-attention manages the integration of inter-modal features, leading to a unified feature representation suitable for downstream tasks. In Section “Emotion–cause pair extraction with graph attention networks”, we introduce the use of GATs for emotion–cause pair extraction. In this stage, the multimodal feature representations are transformed into graph structures, where each node represents a specific segment of data. The GATs are then employed to model the dependencies between these nodes, allowing the system to capture relational information across the multimodal inputs. This mechanism enhances the model’s ability to extract emotion–cause pairs by focusing on interactions between different segments of data within and across modalities. Section “Transformer and GAT Hybrid for Emotion–Cause Detection” describes a hybrid approach combining Transformer architectures with GATs. Transformers contribute global contextual understanding by modelling dependencies between input tokens using self-attention, while GATs enhance the model’s capability to focus on local relational information between graph nodes. This hybrid approach benefits from the complementary strengths of both architectures, leading to improved performance in emotion–cause detection. Finally, Section “Combining outputs using attention mechanism” presents a mechanism to combine the outputs of the Transformer and GAT models using attention. This combination ensures that the most relevant features from both models are dynamically weighted and integrated. A hierarchical structure is adopted to process the outputs from each model independently, followed by merging them using an attention mechanism. This step ensures that the final output captures both the global and local dependencies essential for detecting emotion–cause pairs effectively.

### Multimodal feature extraction

This sub-section describes our approach to extracting features from multiple modalities: text, audio, and video. Each modality is processed independently to capture relevant information before alignment and integration for further analysis. In Section “Text processing” features are extracted from text using BERT (Bidirectional Encoder Representations from Transformers), for audio in Section “Audio processing” we have employed Wav2Vec. Finally in Section “Video processing”, ViT is used for feature extraction from video. In this subsection, we have assumed that all extracted features converge, the proof of this is provided in the Annexure (Lemma 1, Transformer Feature Extraction Convergence), and can be read for further understanding.

#### Text processing

In our approach, textual data is processed using pre-trained transformer models such as BERT^14^. The primary objective of text processing is to extract rich contextual embeddings that encode the semantics of the input sentences while maintaining temporal coherence. Let the input text be represented as a sequence of words or tokens:

1 $$\begin{aligned} X = \{x_1, x_2, x_3, \dots , x_n\} \end{aligned}$$

Where $x_i$ represents the $i$-th token in the sequence, and $n$ is the total number of tokens. Each token is then passed through a BERT model to obtain contextual embeddings. Specifically, for each token $x_i$, we derive a hidden state $h_i$ from the BERT model, such that:

2 $$\begin{aligned} h_i = \text {BERT}(x_i, X) \end{aligned}$$

Where $h_i \in \mathbb {R}^d$ and $d$ is the dimension of the embedding space. The function $\text {BERT}(x_i, X)$ encodes the token $x_i$ in the context of the entire sequence $X$, utilizing the self-attention mechanism inherent to transformer architectures. BERT employs a multi-head self-attention mechanism that allows the model to focus on different parts of the sentence simultaneously. The self-attention score for token $x_i$ attending to token $x_j$ is computed as:

3 $$\begin{aligned} \text {Attention}(x_i, x_j) = \text {softmax}\left( \frac{(h_i W_Q)(h_j W_K)^T}{\sqrt{d_k}} \right) \end{aligned}$$

Where $W_Q$ and $W_K$ are learned projection matrices that project the hidden states $h_i$ and $h_j$ into query and key vectors, respectively, and $d_k$ is the dimension of these vectors. The attention mechanism dynamically weighs the importance of different tokens in the sequence. After applying multi-head attention across the sequence, the model outputs contextual embeddings:

4 $$\begin{aligned} H = \{h_1, h_2, h_3, \dots , h_n\} \end{aligned}$$

Where $H \in \mathbb {R}^{n \times d}$ represents the matrix of contextual embeddings for the entire sequence.

#### Audio processing

In our approach, audio data is processed using pre-trained models such as Wav2Vec. The objective is to extract meaningful features from raw audio signals, which are critical for capturing the nuances of spoken content, including emotional and prosodic variations. Let the raw audio signal be represented as a continuous time-domain signal:

5 $$\begin{aligned} A(t) = \{a_1, a_2, a_3, \dots , a_T\} \end{aligned}$$

Where $A(t)$ is the audio waveform sampled at time $t$, and $T$ represents the total number of time steps in the signal. Each sample $a_i$ corresponds to the amplitude of the audio signal at time step $t_i$. The Wav2Vec model is used to extract high-level audio features from raw waveforms. Initially, the input waveform is segmented into overlapping frames using a sliding window approach, with each frame containing a fixed number of samples. Let $\textbf{f}_i \in \mathbb {R}^k$ represent the $i$-th frame, where $k$ is the window size. Thus, the audio signal can be segmented as:

6 $$\begin{aligned} A(t) = \{\textbf{f}_1, \textbf{f}_2, \textbf{f}_3, \dots , \textbf{f}_T\} \end{aligned}$$

Where $T$ is the total number of frames. Each frame $\textbf{f}_i$ is then passed through the convolutional layers of the Wav2Vec model to capture local features. The output of the convolutional layers, $\textbf{z}_i$, represents the latent speech representation at frame $i$, given by:

7 $$\begin{aligned} \textbf{z}_i = \text {ConvLayers}(\textbf{f}_i) \end{aligned}$$

Where $\textbf{z}_i \in \mathbb {R}^d$, and $d$ is the dimensionality of the latent feature space. These features capture both phonetic and prosodic information from the audio frames. Wav2Vec employs a multi-layer transformer architecture to learn contextualized representations from the latent features $\textbf{z}_i$. For each frame $i$, the transformer processes the latent feature $\textbf{z}_i$ along with its neighbouring frames to capture temporal dependencies. Let $H = \{\textbf{h}_1, \textbf{h}_2, \dots , \textbf{h}_N\}$ represent the sequence of hidden states from the transformer:

8 $$\begin{aligned} \textbf{h}_i = \text {TransformerLayer}(\textbf{z}_i, \textbf{z}_{i-1}, \dots , \textbf{z}_{i+k}) \end{aligned}$$

Where each $\textbf{h}_i \in \mathbb {R}^d$ encodes the contextual information for frame $i$, considering both past and future frames within a context window of size $k$. The self-attention mechanism in the transformer computes attention scores between different frames to determine the contribution of each frame to the contextual representation. For two frames $i$ and $j$, the attention score is computed as:

9 $$\begin{aligned} \text {Attention}(i, j) = \text {softmax}\left( \frac{(\textbf{h}_i W_Q)(\textbf{h}_j W_K)^T}{\sqrt{d_k}} \right) \end{aligned}$$

Where $W_Q$ and $W_K$ are learnable projection matrices for the query and key vectors, and $d_k$ is the dimension of these vectors. The attention mechanism dynamically weighs the importance of different frames based on their relevance to the target frame. The output from the transformer’s final layer is a sequence of contextualized audio embeddings:

10 $$\begin{aligned} H' = \{\textbf{h}'_1, \textbf{h}'_2, \dots , \textbf{h}'_N\} \end{aligned}$$

Where $H' \in \mathbb {R}^{N \times d}$ represents the audio embeddings that capture both local frame-level features and long-range temporal dependencies. These embeddings are then passed to the downstream tasks, such as emotion and cause extraction, allowing the model to integrate the audio modality effectively with other modalities like text and video.

**Algorithm 1:**
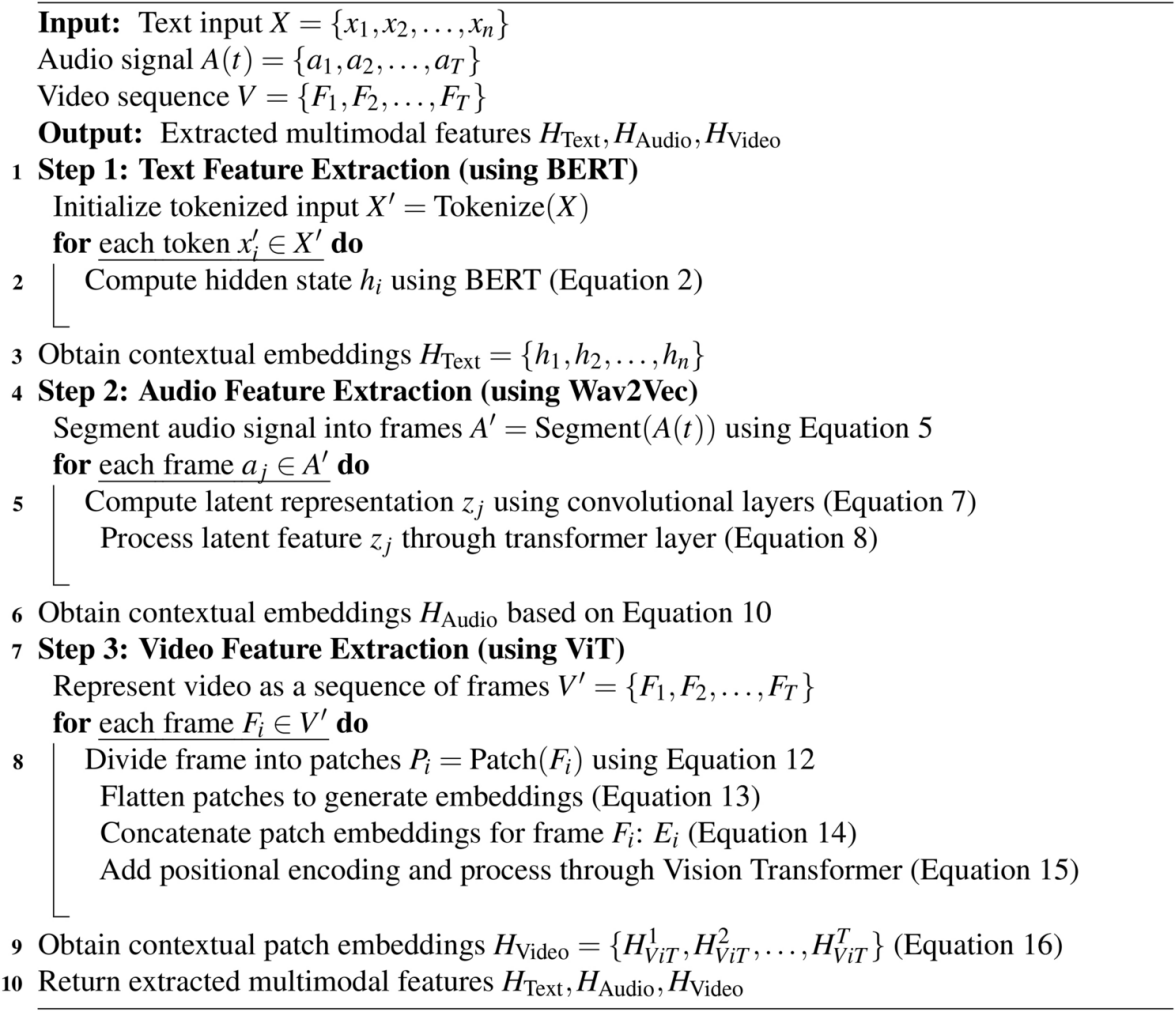
Multimodal feature extraction for text, audio, and video.

**Fig. 2:**
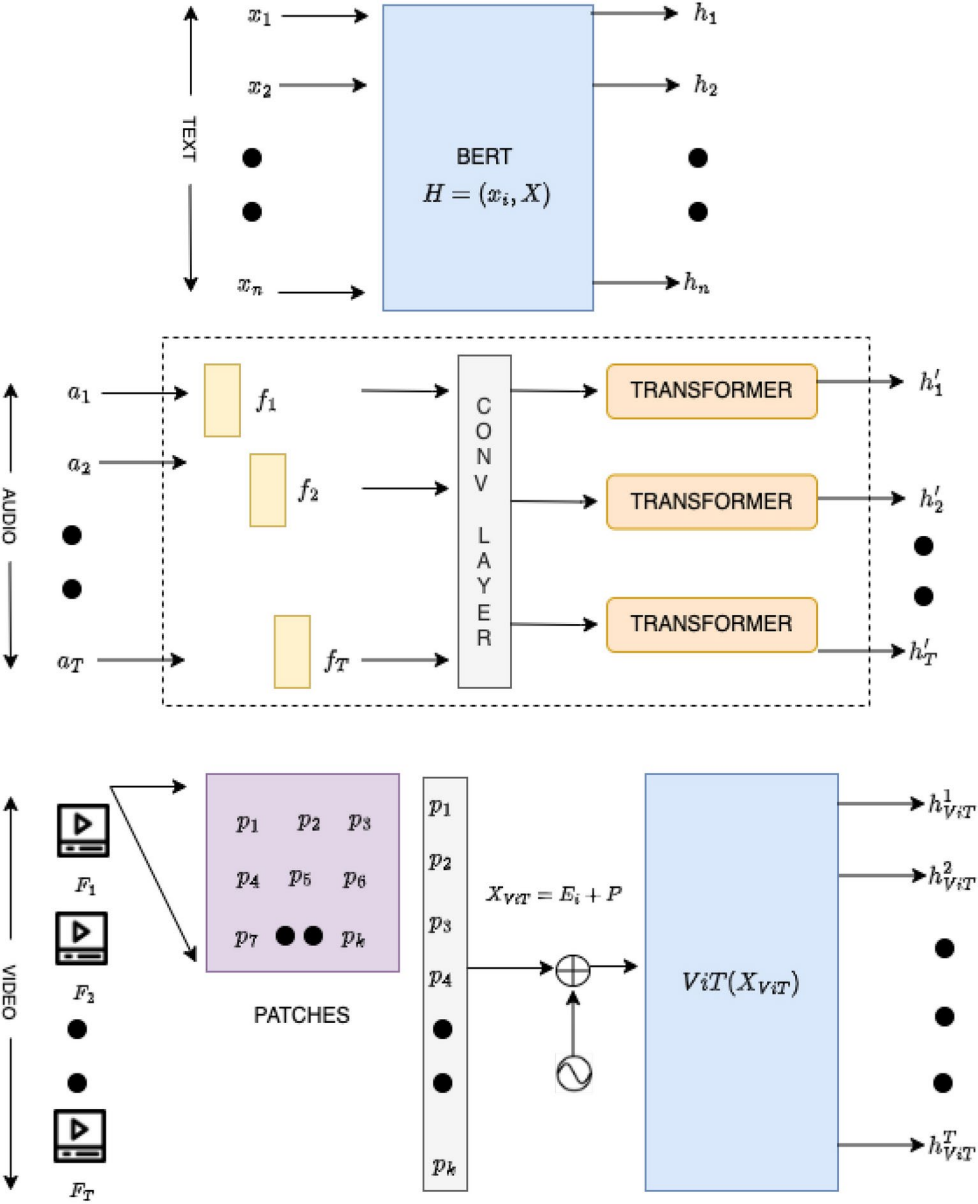
Multimedia feature extraction.

#### Video processing

To extract meaningful visual features from video frames, we utilize ViTs. These transformers operate on image patches, processing temporal and spatial relationships within the video to capture both frame-wise and sequence-based information. Let the video be represented as a sequence of frames:

11 $$\begin{aligned} V = \{F_1, F_2, \dots , F_T\} \end{aligned}$$

Where $F_i$ is the $i$-th video frame, and $T$ is the total number of frames. Each frame $F_i \in \mathbb {R}^{H \times W \times C}$ represents an image with height $H$, width $W$, and $C$ color channels. Each video frame $F_i$ is divided into non-overlapping patches of fixed size $p \times p$, yielding a sequence of image patches:

12 $$\begin{aligned} F_i = \{\textbf{p}_1, \textbf{p}_2, \dots , \textbf{p}_k\} \end{aligned}$$

Where $\textbf{p}_j \in \mathbb {R}^{p \times p \times C}$ represents the $j$-th patch, and $k = \frac{H \times W}{p^2}$ is the total number of patches per frame. These patches are then flattened into 1D vectors, producing patch embeddings:

13 $$\begin{aligned} \textbf{e}_j = \text {Flatten}(\textbf{p}_j) \end{aligned}$$

Where $\textbf{e}_j \in \mathbb {R}^{p^2 \times C}$ represents the flattened patch embedding. The patch embeddings for each frame $F_i$ can be concatenated to form a sequence of embeddings:

14 $$\begin{aligned} E_i = \{\textbf{e}_1, \textbf{e}_2, \dots , \textbf{e}_k\} \end{aligned}$$

Where $E_i \in \mathbb {R}^{k \times (p^2 \times C)}$. These embeddings are then augmented with positional encodings to retain spatial information before being fed into the ViT.

**Fig. 3:**
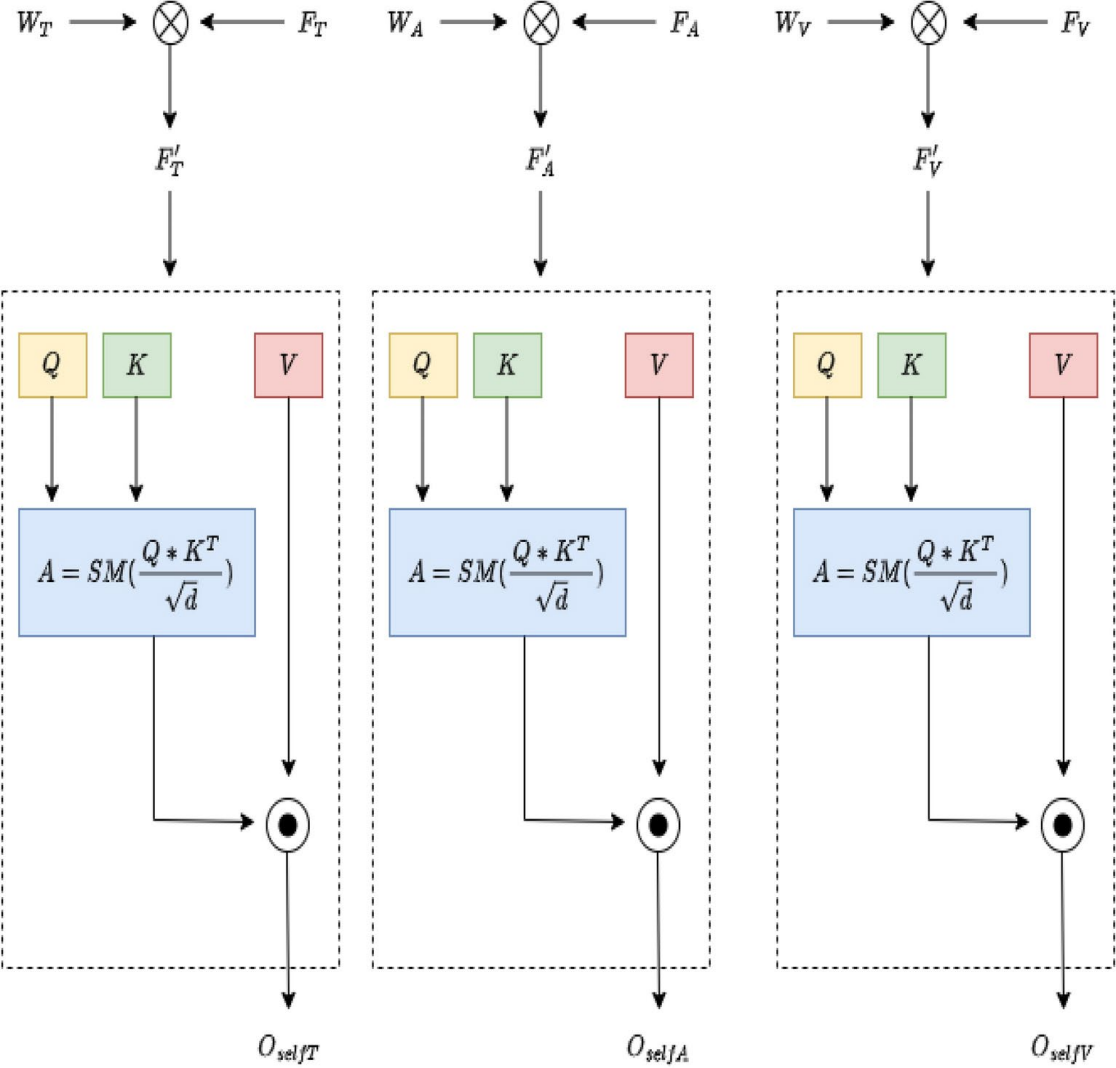
Self attention.

The input to the ViT can be expressed as:

15 $$\begin{aligned} X_{ViT} = E_i + P \end{aligned}$$

Where $P$ represents the positional encoding matrix. The Vision Transformer employs a self-attention mechanism similar to that of text transformers, allowing it to learn relationships between different patches in a frame as well as across frames in a video sequence as depicted in Figure 2. The output of the ViT for frame $i$ can be denoted as:

16 $$\begin{aligned} H_{ViT}^i = \text {ViT}(X_{ViT}) \end{aligned}$$

Where $H_{ViT}^i \in \mathbb {R}^{k \times d}$ represents the contextualized patch embeddings. In Algorithm 1 multimodal feature extraction for text, audio and video is summarized.

### Feature fusion using transformers

Feature fusion is a pivotal process in multi-modal learning, aimed at effectively combining diverse feature sets to enhance the model’s overall performance. Transformers, with their robust self-attention and cross-attention mechanisms, excel in capturing complex dependencies both within individual feature sets and across different modalities. This section elaborates on a novel approach to feature fusion utilizing these attention mechanisms.

#### Input feature representation

Let $\textbf{F}_T \in \mathbb {R}^{n_1 \times d_1}$, $\textbf{F}_A \in \mathbb {R}^{n_2 \times d_2}$, and $\textbf{F}_V \in \mathbb {R}^{n_3 \times d_3}$ represent the text, audio, and video feature sets, respectively. Here, $n_1$, $n_2$, and $n_3$ denote the number of feature vectors, while $d_1$, $d_2$, and $d_3$ indicate their respective dimensions. The goal of feature fusion is to generate a unified representation $\textbf{F}_{\text {fused}} \in \mathbb {R}^{n \times d}$, where $n = n_1 + n_2 + n_3$ and *d* is the selected dimensionality for the fused representation.

To achieve this, we first project the feature sets into a common-dimensional space. Let $\textbf{W}_T \in \mathbb {R}^{d_1 \times d}$, $\textbf{W}_A \in \mathbb {R}^{d_2 \times d}$, and $\textbf{W}_V \in \mathbb {R}^{d_3 \times d}$ be the projection matrices. The projected features are computed as follows:

17 $$\begin{aligned} \textbf{F}_T' = \textbf{F}_T \textbf{W}_T, \quad \textbf{F}_A' = \textbf{F}_A \textbf{W}_A, \quad \textbf{F}_V' = \textbf{F}_V \textbf{W}_V \end{aligned}$$

Where $\textbf{F}_T' \in \mathbb {R}^{n_1 \times d}$, $\textbf{F}_A' \in \mathbb {R}^{n_2 \times d}$, and $\textbf{F}_V' \in \mathbb {R}^{n_3 \times d}$ represent the transformed feature sets.

**Fig. 4:**
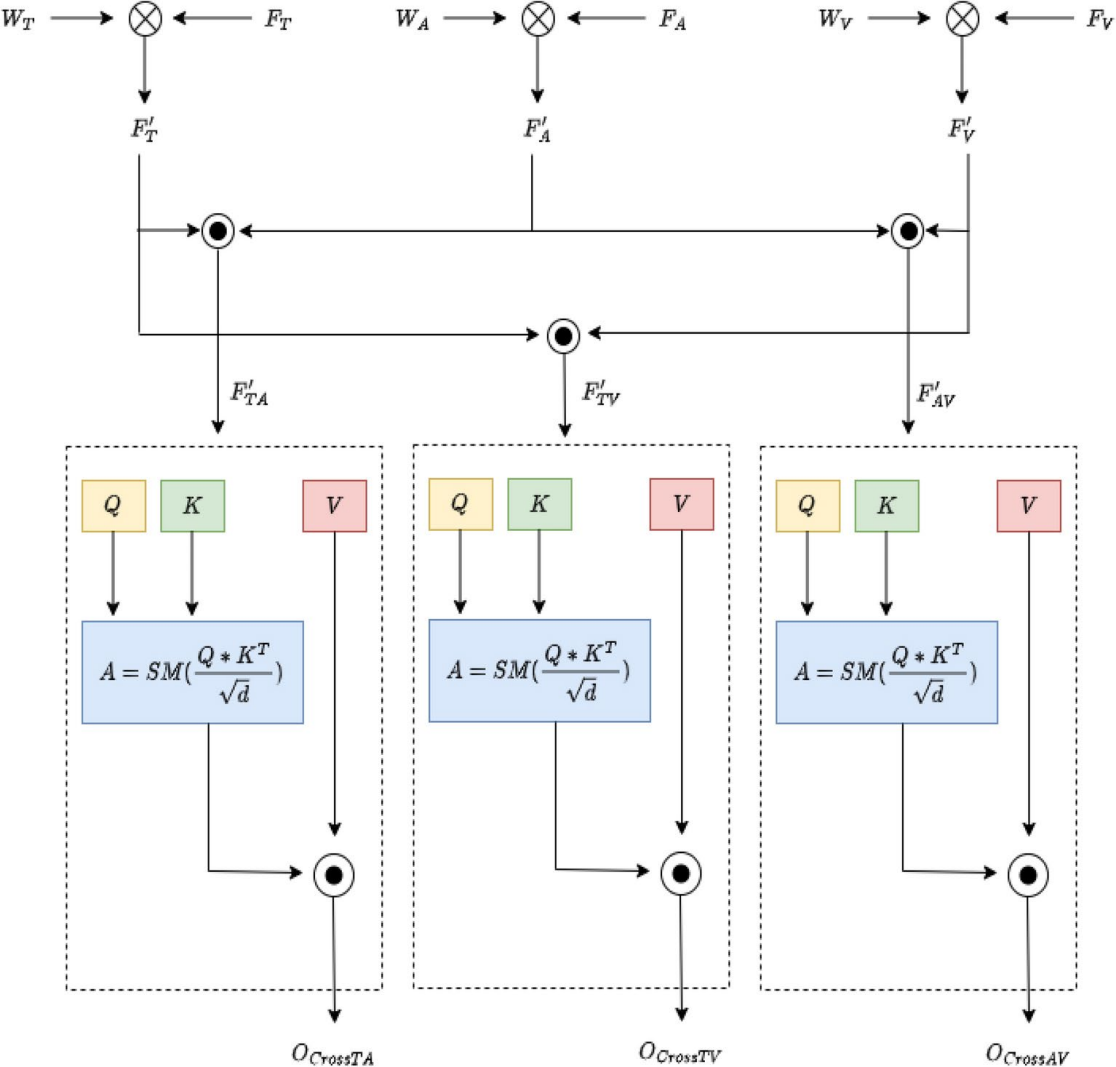
Cross attention.

#### Self-attention mechanism

The self-attention mechanism is fundamental in transformers, allowing the model to weigh the importance of different feature vectors within the same modality. Given a query matrix $\textbf{Q}$, a key matrix $\textbf{K}$, and a value matrix $\textbf{V}$ derived from the same set of features, the attention scores are computed as follows:

18 $$\begin{aligned} \mathbf {A_{Self}} = \text {softmax} \left( \frac{\textbf{Q} \textbf{K}^\top }{\sqrt{d}} \right) \end{aligned}$$

Where $\textbf{A} \in \mathbb {R}^{n \times n}$ is the attention matrix, and *d* is the dimension of the features used for scaling as shown in Figure 3. The output of the self-attention layer is then obtained by:

19 $$\begin{aligned} \textbf{O}_{\text {self}} = \textbf{A} \textbf{V} \end{aligned}$$

This mechanism enables the model to capture relationships and dependencies among different features in the same modality, thus enhancing the representational power of the feature set.

#### Cross-attention for inter-modal fusion

In addition to self-attention, cross-attention can be employed to fuse features from different modalities. For instance, let $\textbf{Q}$ be derived from the text features $\textbf{F}_T'$ while $\textbf{K}$ and $\textbf{V}$ are derived from the audio features $\textbf{F}_A'$:

20 $$\begin{aligned} \textbf{A}_{\text {cross}} = \text {softmax} \left( \frac{\textbf{Q} \textbf{K}^\top }{\sqrt{d}} \right) \end{aligned}$$

The output of the cross-attention layer is given by:

21 $$\begin{aligned} \textbf{O}_{\text {cross}} = \textbf{A}_{\text {cross}} \textbf{V} \end{aligned}$$

In this case, the model learns how to align the textual features with the audio features, allowing for a better representation of the underlying relationships across modalities as shown in Figure 4. This process can be similarly applied for audio and video, or text and video pairs, creating a rich, interconnected representation.

#### Feature aggregation

To aggregate the outputs from both self-attention and cross-attention, we concatenate the results:

22 $$\begin{aligned} \textbf{O}_{\text {combined}} = \text {concat}(\textbf{O}_{\text {self}}, \textbf{O}_{\text {cross}}) \end{aligned}$$

This combined output is then passed through a feed-forward neural network (FFN):

23 $$\begin{aligned} \textbf{F}_{\text {fused}} = \text {LayerNorm}\left( \textbf{O}_{\text {combined}} + \text {FFN}(\textbf{O}_{\text {combined}}) \right) \end{aligned}$$

The FFN is defined as follows:

24 $$\begin{aligned} \text {FFN}(\textbf{X}) = \text {ReLU}(\textbf{X} \textbf{W}_1 + \textbf{b}_1) \textbf{W}_2 + \textbf{b}_2 \end{aligned}$$

Where $\textbf{W}_1 \in \mathbb {R}^{d \times d_{\text {hidden}}}$ and $\textbf{W}_2 \in \mathbb {R}^{d_{\text {hidden}} \times d}$ are the weights of the FFN, and $\textbf{b}_1$ and $\textbf{b}_2$ are the bias terms. This structure allows for the learned representations to be fine-tuned further, ensuring that the combined features are both coherent and informative for downstream tasks. Through these mechanisms, the proposed approach effectively captures both intra-modal dependencies via self-attention and inter-modal relationships through cross-attention, thereby enriching the fused feature representation for improved performance in multi-modal tasks.

### Emotion–cause pair extraction with graph attention networks

Emotion–cause pair extraction is a key task in understanding the underlying triggers of emotions. This task is particularly challenging when dealing with multi-modal data (e.g., text, audio, video). To tackle this complexity, GATs can be employed to capture relationships between different segments of input data. GATs can model dependencies between nodes in a graph, using an attention mechanism to weigh the relevance of connections between nodes. In this subsection, we describe the use of GATs for modelling interactions between multi-modal data segments and how these interactions can help extract emotion–cause pairs. We have used the node stability assumption for embedding without proofing it, the formal proof of it can be found in the Annexure of the paper (Lemma 2, GAT Node Embedding Stability). We have also assumed that the attention scores are normalized which is proved in the Annexure of the paper (Lemma 3, Attention Score Normalization).

#### Graph construction

After fusing multi-modal features (combining text, audio, and video data), we construct a graph to represent relationships between these features. In this graph, **nodes** correspond to different segments of the input, such as words in text, frames in video, or segments of audio. The **edges** represent relationships between these segments, such as syntactic dependencies, temporal proximity, or semantic similarity. Let the input multi-modal features be represented as:

25 $$\begin{aligned} X = \{x_1, x_2, \dots , x_n\}, \end{aligned}$$

where $x_i$ is the feature vector corresponding to the $i$-th segment of input data. These features are derived from text, audio, and video modalities.

**Algorithm 2:**
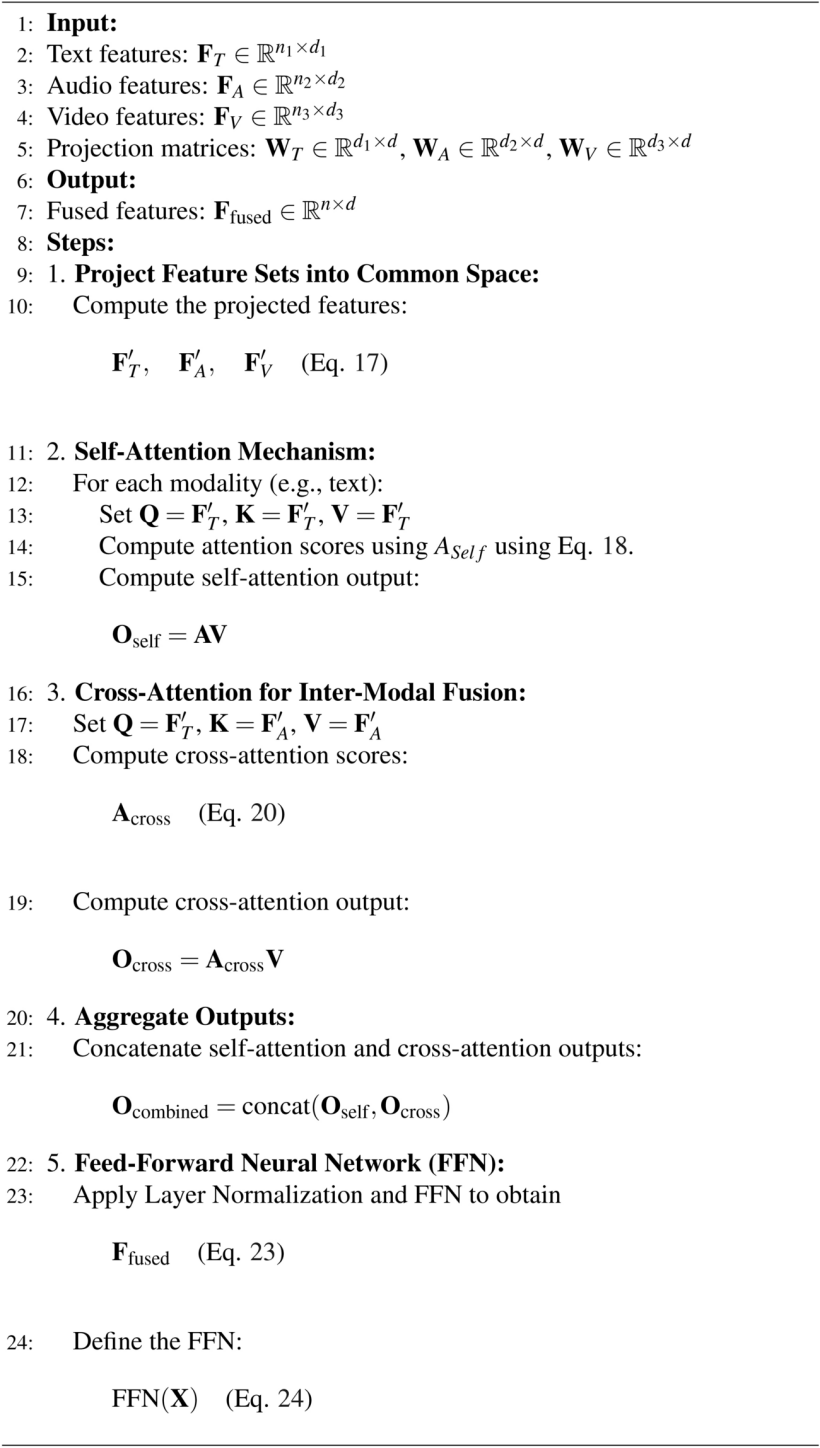
Feature fusion using transformers.

The input data is represented as a graph $G = (V, E)$, where $V = \{v_1, v_2, \dots , v_n\}$ are the nodes, and $E \subseteq V \times V$ are the edges between nodes. The adjacency matrix $A$ encodes the edges:

26 $$\begin{aligned} A_{ij} = {\left\{ \begin{array}{ll} 1 & \text {if there is an edge between } v_i \text { and } v_j, \\ 0 & \text {otherwise.} \end{array}\right. } \end{aligned}$$

The graph captures both temporal and syntactic relationships across modalities.

#### GATs for emotion–cause pairing

Once the graph is constructed, GATs are used to model the interactions between different segments. Each node represents a candidate for either an emotion or a cause. The attention mechanism in GATs allows the model to focus on the most important connections between nodes, learning which nodes (i.e., emotions and causes) are related. For each node $v_i$, the feature vector $h_i$ is updated based on its neighbors $v_j$. The attention score $\alpha _{ij}$ between node $v_i$ and its neighbor $v_j$ is computed as:

27 $$\begin{aligned} \alpha _{ij} = \frac{\exp \left( \text {LeakyReLU} \left( \textbf{a}^\top \left[ \textbf{W} h_i \parallel \textbf{W} h_j \right] \right) \right) }{\sum _{k \in \mathcal {N}(i)} \exp \left( \text {LeakyReLU} \left( \textbf{a}^\top \left[ \textbf{W} h_i \parallel \textbf{W} h_k \right] \right) \right) }, \end{aligned}$$

Where $\textbf{a}$ is a learnable attention vector, $\textbf{W}$ is a learnable weight matrix, and $[ \cdot \parallel \cdot ]$ represents concatenation. The softmax function ensures that attention scores $\alpha _{ij}$ are normalized across all neighbours of the node $v_i$. The node’s feature vector $h'_i$ is updated by aggregating its neighbours’ features, weighted by the attention scores:

28 $$\begin{aligned} h'_i = \sigma \left( \sum _{j \in \mathcal {N}(i)} \alpha _{ij} \textbf{W} h_j \right) , \end{aligned}$$

Where $\sigma (\cdot )$ is a non-linear activation function, such as ReLU.

#### Emotion–cause relationship extraction

The attention mechanism in GATs is used to calculate the relevance of each node in the graph to potential emotions or causes. Let $v_i$ represent an emotion node and $v_j$ represent a cause node. If the attention score $\alpha _{ij}$ is above a certain threshold $\tau$, the pair $(v_i, v_j)$ is considered an emotion–cause pair:

29 $$\begin{aligned} (e, c) = \{(v_i, v_j) \, | \, \alpha _{ij} > \tau \}, \end{aligned}$$

Where $\tau$ is the threshold for significant attention scores. The relationships may span across different modalities. For example, an emotion detected from the text may be caused by an event captured in a video. GATs model these cross-modal relationships by allowing edges between nodes representing different modalities. GATs enable the incorporation of both temporal and syntactic relationships. For example, in video data, the cause of an emotion may occur several frames before the emotion is expressed. Similarly, in text, syntactic dependencies can be modelled between words. The adjacency matrix $A$ can thus be modified to account for these relationships:

30 $$\begin{aligned} A_{ij} = {\left\{ \begin{array}{ll} 1 & \text {if nodes } v_i, v_j \text {temporally/ syntactically related}, \\ 0 & \text {otherwise}. \end{array}\right. } \end{aligned}$$

By constructing a graph of multi-modal input data and using GATs, we can effectively model relationships between segments and identify emotion–cause pairs. The attention mechanism in GATs allows selective focus on the most important connections, leading to accurate emotion–cause pair extraction. Incorporating both temporal and syntactic relationships allows for a comprehensive understanding of emotion–cause dependencies.

**Algorithm 3:**
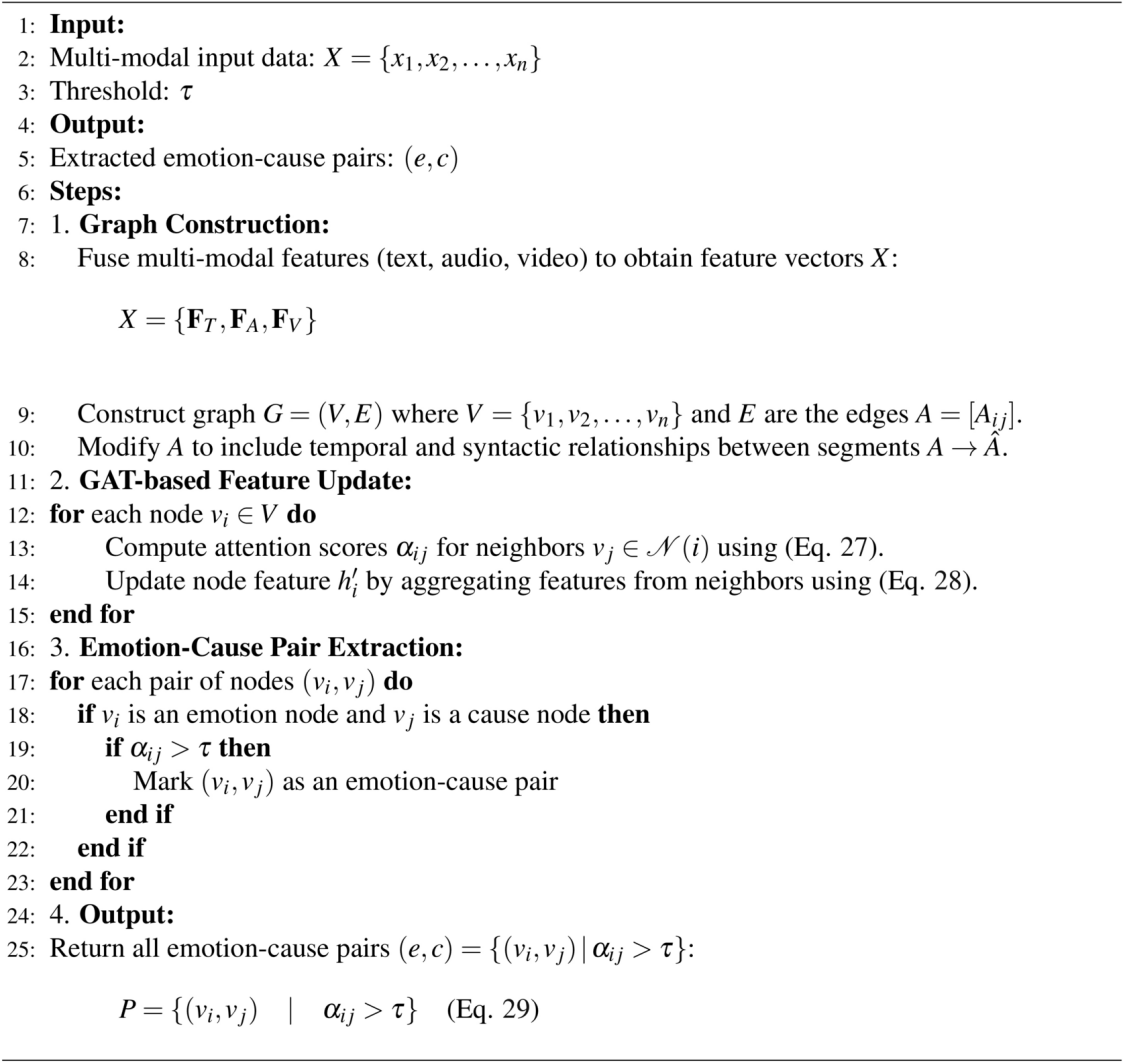
Emotion–cause pair extraction using GATs.

### Transformer and GAT Hybrid for emotion–cause detection

The integration of Transformer architectures with GATs provides a powerful framework for detecting emotion–cause pairs within multi-modal data. This hybrid approach leverages the strengths of both architectures: the global context understanding of Transformers and the localized relational modelling of GATs. In this section, we outline how this combination can be effectively utilized for emotion–cause detection, focusing on the mathematical formulations involved in both components. Transformers utilize self-attention mechanisms to model dependencies between input tokens, allowing for the capture of contextual information regardless of their positions. Given a sequence of input embeddings $E = \{e_1, e_2, \ldots , e_n\}$, the self-attention mechanism computes the attention scores $A$ as follows:

31 $$\begin{aligned} A_{ij} = \frac{\exp \left( \frac{Q_i K_j^\top }{\sqrt{d_k}}\right) }{\sum _{k=1}^n \exp \left( \frac{Q_i K_k^\top }{\sqrt{d_k}}\right) }, \end{aligned}$$

Where $Q_i$, $K_j$, and $V_j$ represent the query, key, and value embeddings for the $i$-th and $j$-th tokens respectively, and $d_k$ is the dimension of the key vectors. The output of the self-attention mechanism for each input embedding can be calculated as:

32 $$\begin{aligned} \text {Output}_i = \sum _{j=1}^n A_{ij} V_j. \end{aligned}$$

This mechanism allows the Transformer to focus on relevant tokens within the input sequence, which is crucial for understanding emotions in context. In our hybrid architecture, after obtaining the contextual embeddings from the Transformer, we can construct a graph similar to the previous sections. The transformed embeddings $H = \{h_1, h_2, \ldots , h_n\}$ from the Transformer are utilized as the initial node features for GAT processing. Each node $v_i$ in the graph corresponds to a transformed embedding $h_i$, which is then updated based on the relationships defined by the graph structure. The attention scores $\alpha _{ij}$ between nodes $v_i$ and $v_j$ can be computed using the updated feature vectors $h'_i$ and $h'_j$:

33 $$\begin{aligned} \alpha _{ij} = \frac{\exp \left( \text {LeakyReLU}\left( \textbf{a}^\top \left[ \textbf{W} h'_i \parallel \textbf{W} h'_j\right] \right) \right) }{\sum _{k \in \mathcal {N}(i)} \exp \left( \text {LeakyReLU}\left( \textbf{a}^\top \left[ \textbf{W} h'_i \parallel \textbf{W} h'_k\right] \right) \right) }. \end{aligned}$$

This formula integrates the learned representations from the Transformer into the GAT framework, allowing the model to focus on the most relevant interactions between segments.

**Algorithm 4:**
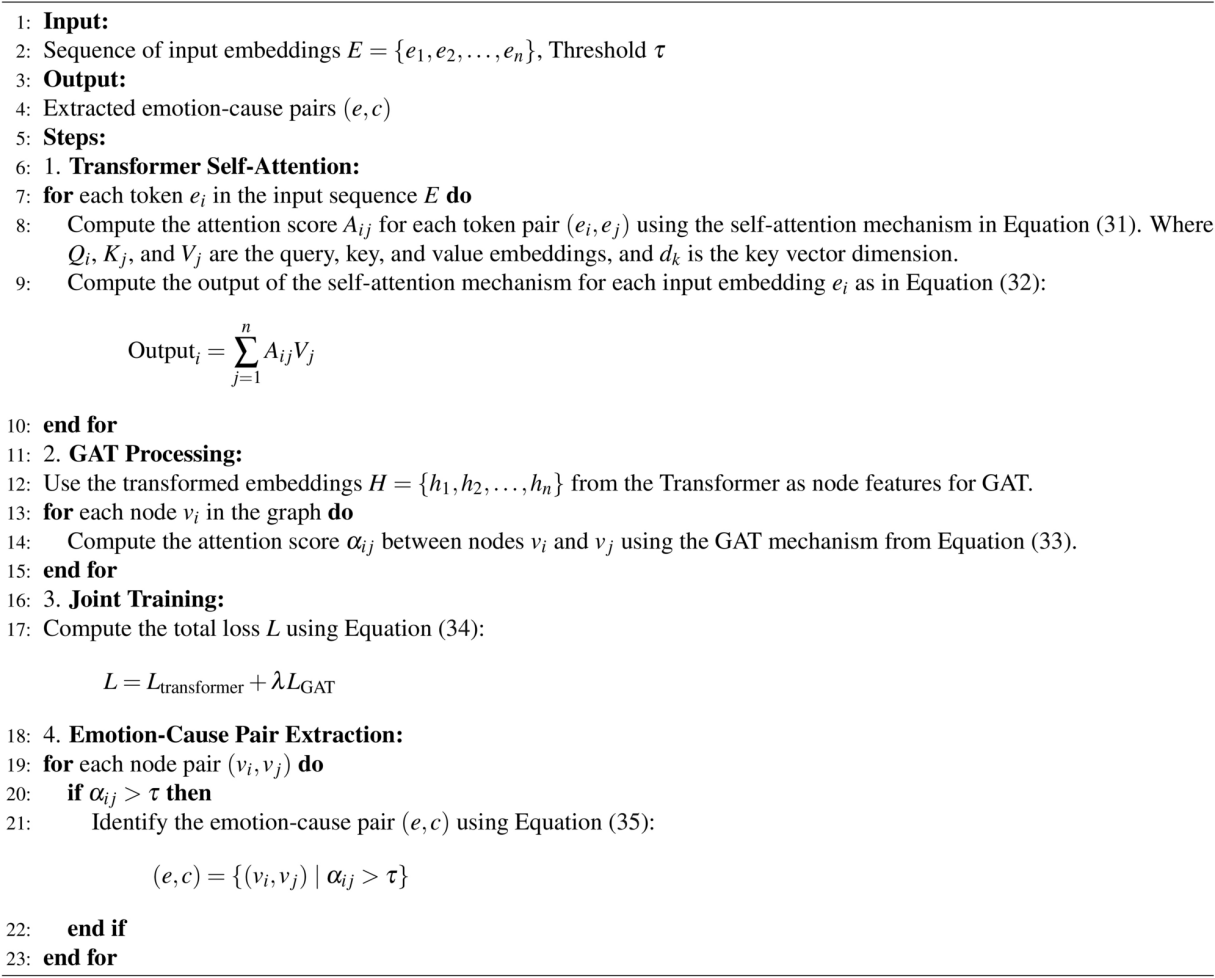
Transformer and GAT hybrid for emotion–cause detection.

#### Joint training for emotion–cause detection

To optimize the detection of emotion–cause pairs, we employ a joint training strategy. The overall loss $L$ can be formulated as a combination of two losses: the loss from the Transformer and the GAT loss, given by:

34 $$\begin{aligned} L = L_{\text {transformer}} + \lambda L_{\text {GAT}}, \end{aligned}$$

Where $\lambda$ is a hyperparameter that balances the contributions of both components. The Transformer loss $L_{\text {transformer}}$ can be derived from cross-entropy based on the predicted emotional states, while the GAT loss $L_{\text {GAT}}$ can be based on the accuracy of the detected emotion–cause pairs.

The emotion–cause relationship extraction follows a similar approach to that described previously. Given the output of the GAT, if the attention score $\alpha _{ij}$ exceeds a threshold $\tau$, the pairs can be identified:

35 $$\begin{aligned} (e, c) = \{(v_i, v_j) \mid \alpha _{ij} > \tau \}. \end{aligned}$$

This framework allows for a robust model capable of processing complex dependencies across different modalities, ultimately improving the performance of emotion–cause pair detection. By integrating Transformers with GATs, we enhance the capability of emotion–cause detection systems to leverage both global contextual relationships and local node interactions. This hybrid model facilitates a deeper understanding of how emotions are triggered by various causes, making it an effective solution for multi-modal emotion analysis.

**Algorithm 5:**
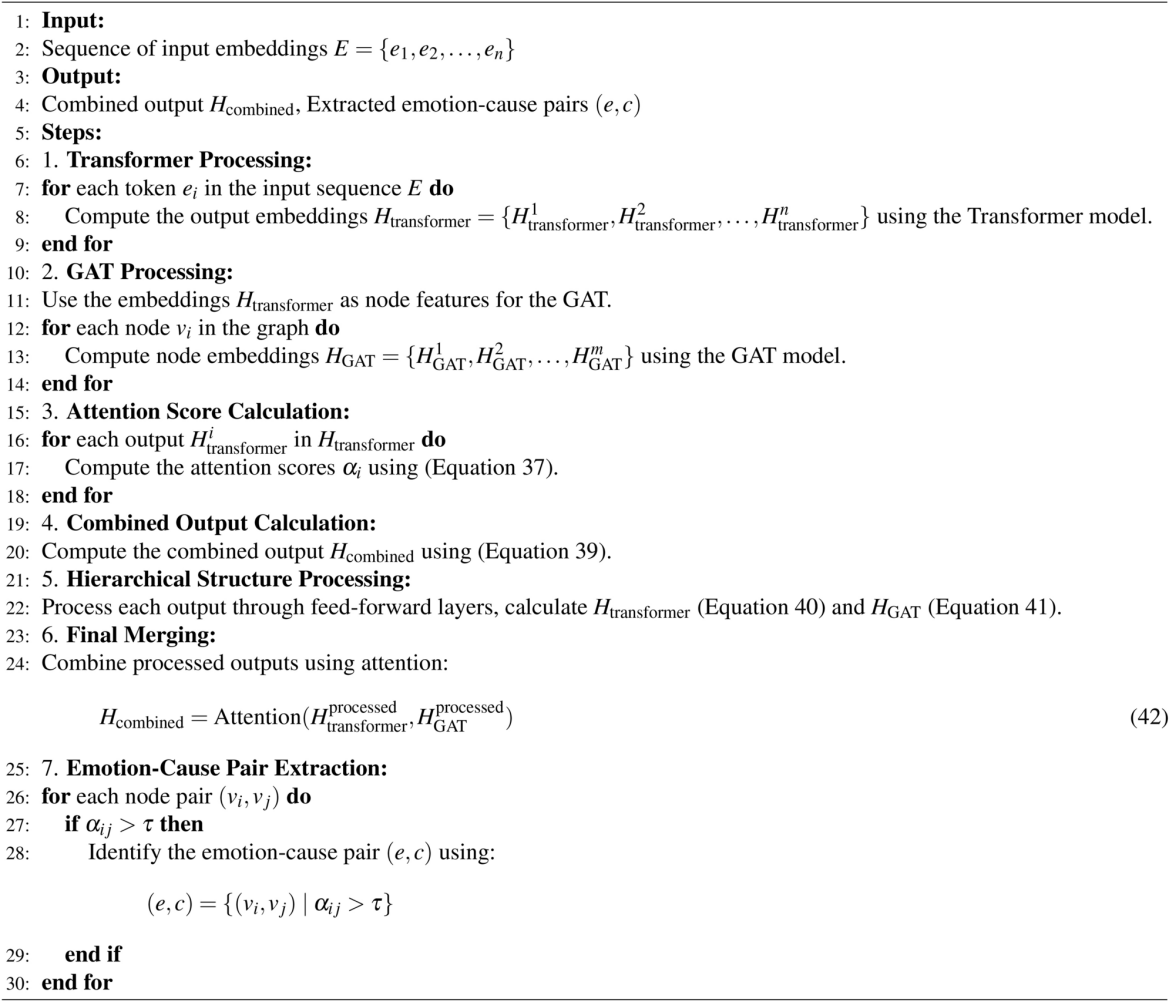
Combining outputs using attention mechanism.

### Combining outputs using attention mechanism

In our proposed hybrid framework, we integrate the outputs from the Transformer and GAT using a final attention mechanism. This mechanism dynamically assigns weights to the outputs from both components, allowing the model to prioritize the most relevant information for emotion–cause detection. The combined output $H_{\text {combined}}$ is defined as follows:

36 $$\begin{aligned} H_{\text {combined}} = \text {Attention}(H_{\text {transformer}}, H_{\text {GAT}}) \end{aligned}$$

In the above equation, we have assumed the correctness and optimality of weighted output can be seen from Lemma 4 and 5 of Annexure. To compute the combined representation, we first calculate the attention scores $\alpha _i$ for each component’s output. The scores reflect the relevance of the $i$-th output from the Transformer concerning the GAT output:

37 $$\begin{aligned} \alpha _i = \frac{\exp \left( \text {score}(H_{\text {transformer}}^i, H_{\text {GAT}})\right) }{\sum _{j=1}^{n} \exp \left( \text {score}(H_{\text {transformer}}^j, H_{\text {GAT}})\right) } \end{aligned}$$

Where the scoring function $\text {score}(\cdot )$ can be defined as the dot product between the $i$-th Transformer output and the GAT output, expressed as:

38 $$\begin{aligned} \text {score}(H_{\text {transformer}}^i, H_{\text {GAT}}) = H_{\text {transformer}}^i \cdot H_{\text {GAT}} \end{aligned}$$

Using these attention scores, we can weight the outputs of both the Transformer and GAT as follows:

39 $$\begin{aligned} H_{\text {combined}} = \sum _{i=1}^{n} \alpha _i H_{\text {transformer}}^i + \sum _{j=1}^{n} (1 - \alpha _j) H_{\text {GAT}}^j \end{aligned}$$

This formulation ensures that $H_{\text {combined}}$ captures the most pertinent features from both modalities, enhancing the model’s capacity to identify emotion–cause pairs effectively.

#### Hierarchical structure

Incorporating a hierarchical structure into our framework further enriches the output combination process. This structure enables independent processing of the Transformer and GAT outputs before merging them. We have assumed that emotion causes a pair to converge proof can be seen in (Lemma 10, Convergence of emotion–cause Detection), similarly, the Uniqueness of the emotion pair is assumed (Lemma 8, Uniqueness of emotion–cause Pair extraction). We define the hierarchical processing as follows:

**Independent Processing**: Each output is passed through separate feed-forward layers, which can be defined mathematically as:
  40 $$\begin{aligned} & H_{\text {transformer}}^{\text {processed}} = \sigma (W_{\text {transformer}} H_{\text {transformer}} + b_{\text {transformer}}) \end{aligned}$$
  41 $$\begin{aligned} & \quad H_{\text {GAT}}^{\text {processed}} = \sigma (W_{\text {GAT}} H_{\text {GAT}} + b_{\text {GAT}}) \end{aligned}$$
  Where $W$ and $b$ are the weights and biases for each layer, and $\sigma$ is an activation function such as ReLU.
**Merging**: After processing, the outputs are combined using the attention mechanism:
  42 $$\begin{aligned} H_{\text {combined}} = \text {Attention}(H_{\text {transformer}}^{\text {processed}}, H_{\text {GAT}}^{\text {processed}}) \end{aligned}$$
  By adopting both the attention mechanism and a hierarchical structure, our model becomes increasingly robust, effectively integrating the global contextual information from the Transformer with the localized relational insights provided by the GAT.

**Fig. 5:**
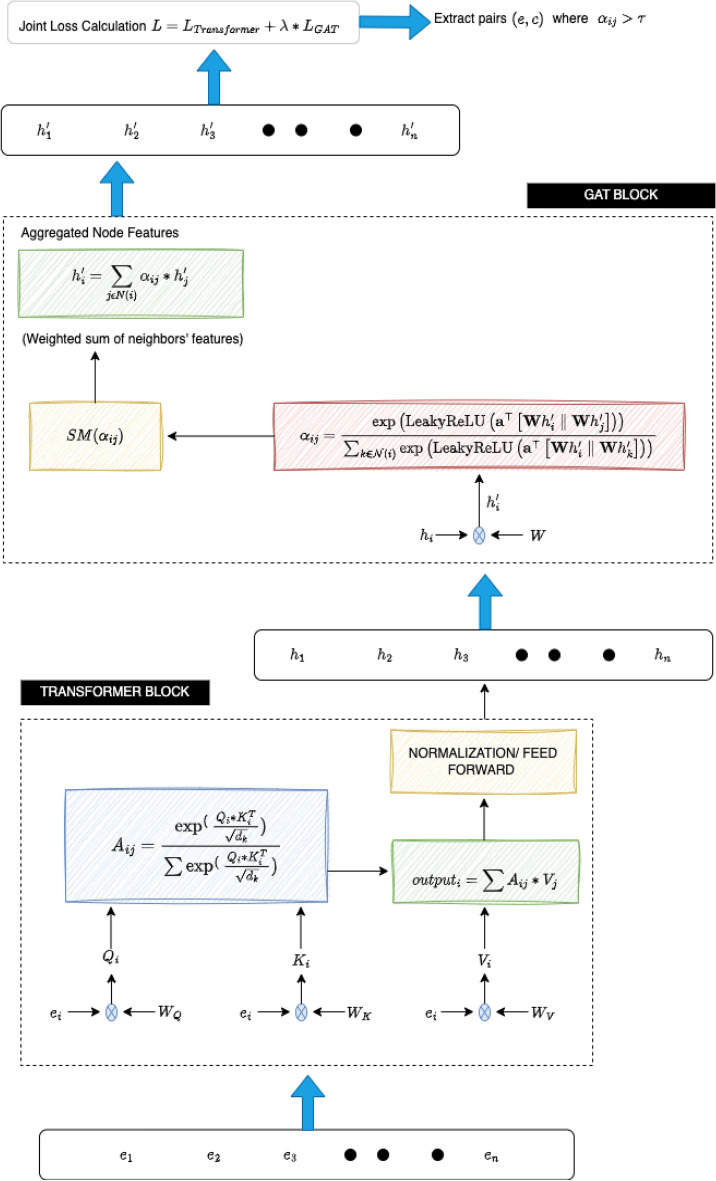
Emotion cause extraction.

The figure 5 represents a hybrid architecture for emotion cause extraction, combining a Transformer Block and GAT Block. The architecture is designed to capture both sequential dependencies (through the Transformer) and graph-based relational information (via the GAT). The bottom part of the figure represents the transformer Block, it processes the input sequence of emotion words $(e_1, e_2, e_3, \dots , e_n)$. Each emotion word is embedded into a vector space and processed through an attention mechanism, which computes interactions between word vectors using query, key, and value matrices.

- **Attention mechanism:** The attention mechanism assigns different weights to each word in the sequence based on their relevance to the current word, this is achieved by computing attention scores.
- **Normalization and feed-forward:** After calculating the attention-weighted sum of the values, the output is normalized and passed through a feed-forward neural network.

The Transformer block produces hidden representations $(h_1, h_2, h_3, \dots , h_n)$ corresponding to each input emotion word. The GAT block is given in the middle of the figure, after obtaining the hidden representations from the Transformer, these are fed into the GAT block.

- **Self-attention in GAT:** Each node in the graph (i.e., each hidden representation $h_i'$) computes attention scores with its neighbouring nodes.
- **Feature aggregation:** Once the attention scores $(\alpha _{ij})$ are computed, the features of neighbouring nodes are aggregated in a weighted sum, where the weights are given by the attention scores. This allows each node to incorporate information from its neighbours.

The GAT block produces an updated set of hidden representations $(h_1', h_2', h_3', \dots , h_n')$, which capture both the node features and their relational information from the graph structure. Finally, the top section of the figure shows Joint Loss Calculation, the architecture employs a joint loss function that combines the losses from both the Transformer block $(L_{\text {Transformer}})$ and the GAT block $(L_{\text {GAT}})$. The overall loss is a weighted sum of these two components, with a weight factor $\lambda$. Based on the attention coefficients $(\alpha _{ij})$, pairs of emotion terms $(e_i, c_j)$ are extracted if their attention weight exceeds a predefined threshold $(\tau )$.

In summary, the proposed hybrid framework for emotion–cause detection combines Transformers and GATs to enhance the detection of emotion–cause pairs in multi-modal data. The integration of these models allows for leveraging both global contextual relationships and local node interactions, improving the system’s performance in complex multi-modal emotion analysis tasks. The framework utilizes a joint training strategy that combines the losses from both the Transformer and GAT components. A hyperparameter is employed to balance the contributions of these two losses. The Transformer loss is derived from cross-entropy based on the predicted emotional states, while the GAT loss evaluates the accuracy of detected emotion–cause pairs. This approach ensures that the model is not only learning to identify emotions but also effectively establishing relationships between emotions and their causes. emotion–cause relationship extraction is achieved by analyzing attention scores from the GAT output. If the attention score exceeds a specific threshold, the corresponding pairs are identified as emotion–cause pairs. This method provides a systematic approach to extracting relevant pairs, allowing the model to handle complex dependencies and improve its accuracy in identifying how emotions are triggered by specific causes. An essential part of this hybrid model is the attention mechanism used to combine outputs from the Transformer and GAT components. The attention mechanism dynamically assigns weights to outputs from both models, prioritizing the most relevant information for emotion–cause detection. The combined output is computed through an attention-based weighting process, which considers the relevance of each component’s outputs. This weighted combination enhances the model’s ability to detect emotion–cause pairs effectively. The proposed framework also incorporates a hierarchical structure, enriching the output combination process. The hierarchical processing involves independently processing outputs from the Transformer and GAT through separate feed-forward layers. These processed outputs are then merged using the attention mechanism, resulting in a robust final output that integrates both global and local information. The model’s hierarchical structure allows for independent processing of the Transformer and GAT outputs before merging. Each output is passed through feed-forward layers, applying activation functions to produce processed representations. The merging process uses the attention mechanism to combine the independently processed outputs, strengthening the model’s capacity to integrate global contextual information from the Transformer with the localized relational insights from the GAT. The figure illustrating the architecture shows how the model processes the input sequence of emotion words through the Transformer Block. This block captures sequential dependencies using an attention mechanism that computes interactions between word vectors using query, key, and value matrices. The attention mechanism assigns weights to words based on their relevance, normalizes the output, and processes it through a feed-forward neural network, resulting in hidden representations for each input word. The GAT Block takes the hidden representations from the Transformer Block and applies self-attention to compute scores with neighbouring nodes. It aggregates features of neighbouring nodes using a weighted sum where the weights are derived from attention scores. This aggregation allows each node to incorporate relational information from the graph structure, enhancing the representation of node features. The joint loss calculation in the architecture uses a combination of the Transformer and GAT losses. The overall loss is a weighted sum of these components, governed by a weighting factor. The architecture extracts emotion–cause pairs based on attention coefficients, selecting pairs whose attention weights exceed a predefined threshold. This method ensures that the model effectively identifies relevant emotion–cause pairs, contributing to a deeper understanding of how emotions are influenced by various causes. The hybrid model’s design is particularly well-suited for multi-modal emotion analysis, offering a versatile and powerful approach to detecting complex emotion–cause relationships. By combining the strengths of Transformers and GATs, the model achieves a robust balance between capturing sequential dependencies and leveraging relational insights, leading to improved performance in identifying emotion–cause pairs across different modalities.

## Results and performance evaluation

This section presents the overall result and performance of the proposed system and compares it with the state of the art. Section “Datasets” discusses the datasets used in this study and the data distribution. The second Section “Performance metrics” provides evaluation metrics for the assessment of results. The third Section “Baseline” provides a baseline for the evaluation of the evaluations. The results are provided in two subsections, emotion detection experimental results are presented in Section “Emotion detection results discussion and SOA comparison” and for cause pair extraction results are given in Section “Analysis of confusion matrices”. A more detailed analysis of the result is given in Section “Emotion cause pair extraction results” which is a confusion matrix, while the ablation study is presented in Section “Ablation study”.

**Fig. 6:**
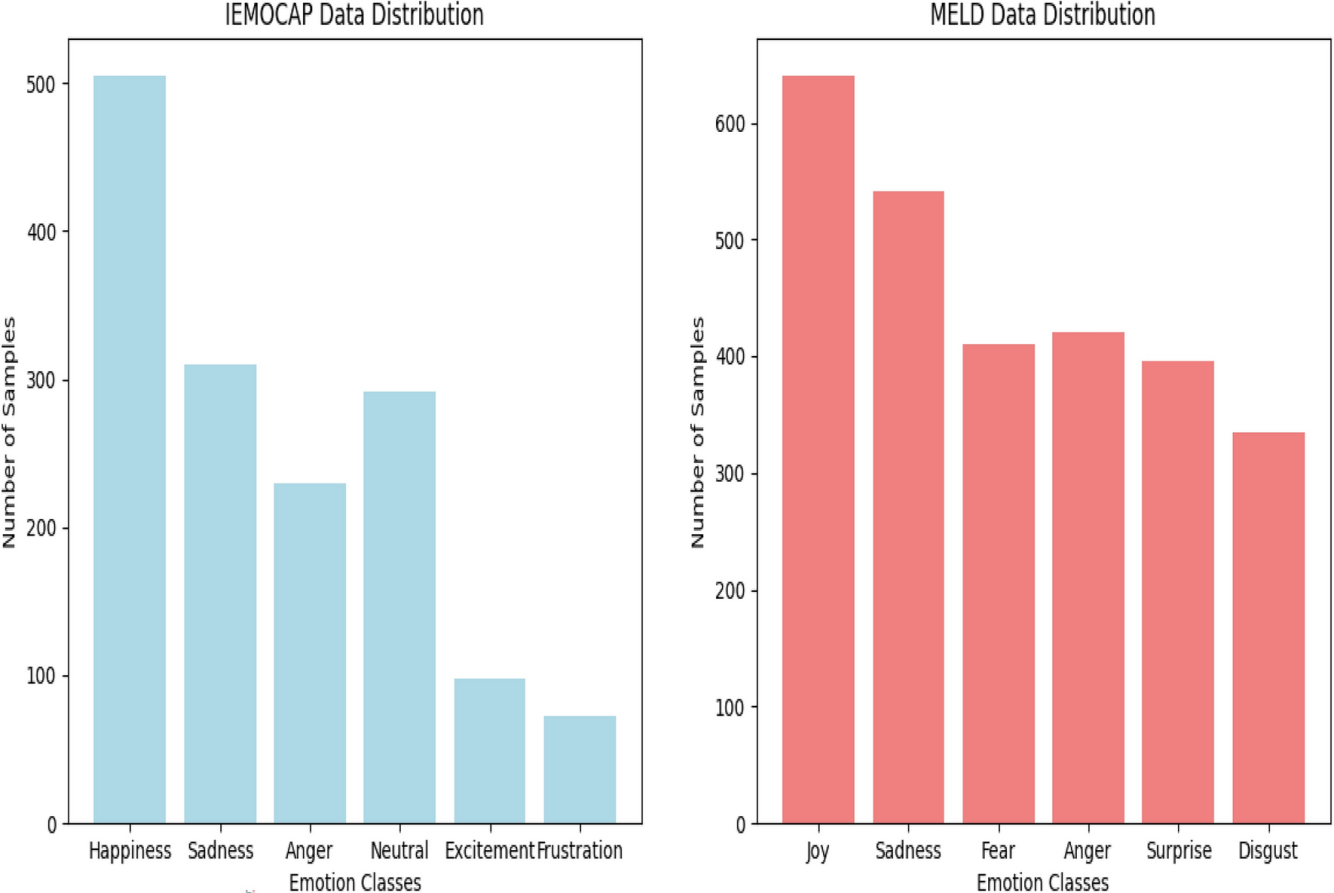
IEMOCAP Vs. MELD.

### Datasets

Our experiments utilized two benchmark datasets for emotion recognition: the Multimodal EmotionLines Dataset (MELD)^4^ and the Interactive Emotional Dyadic Motion Capture Database (IEMOCAP)^46^. MELD (Multimodal EmotionLines Dataset), consists of conversations from the TV show Friends, annotated with emotions in multiple modalities (text, audio, and visual). This dataset not only provides a rich resource for training and evaluating multimodal emotion recognition systems but also highlights the importance of contextual understanding in emotional analysis. The second dataset is CMU-MOSEI (Multimodal Opinion Sentiment and Emotion Intensity), which offers a diverse range of sentences annotated for sentiment and emotion across different modalities. MELD consists of 13,707 conversation clips, where each clip is annotated with one of six emotion labels: joy, sadness, fear, anger, surprise, and disgust. This dataset has been widely used in multimodal emotion recognition tasks due to its large size and inclusion of various emotions. On the other hand, IEMOCAP contains 7,532 samples, each annotated with one of six emotion categories: happiness, sadness, anger, neutral, excitement, and frustration. This dataset focuses on dyadic interactions and is specifically designed for emotion recognition in human dialogue. The dataset is split into 80/20 training and testing, having 1506 and 2741 for testing on IEMOCAP and MELD respectively. Figure 6 shows the distribution of IEMOCAP and MELD for different emotion classes.

We employed two additional datasets for cause-effect pair extraction tasks: ConvECPE and ECF. The ECF dataset contains 151 dialogues comprising 7,433 utterances, making it a valuable resource for exploring cause-effect relations in conversational data.

### Performance metrics

The performance of our model is evaluated using two primary metrics: accuracy (ACC) and weighted F1-score (WF1) for the Multimodal Emotion Recognition Classification (MERC) task, which were also employed by previous studies^18,17,21,23^. In particular, WF1 is used as a more balanced evaluation metric to account for class imbalance. The WF1 score is computed by weighting the F1 scores of individual classes according to the proportion of samples in each class. The formula used to compute the WF1 is as follows:

43 $$\begin{aligned} WF1 = \sum _{i=1}^{n} \left( \frac{\text {class count}_i}{\text {total count}} \times F1_i \right) \end{aligned}$$

In this equation, $F1_i$ represents the F1 score for class $i$, $\text {class count}_i$ is the number of instances in class $i$, $\text {total count}$ refers to the total number of instances across all classes, and $n$ is the number of classes. For the Emotion Cause Pair Extraction (ECPE) task on the ECF and ConvECPE datasets, we employ precision (P), recall (R), and F1-score as the evaluation metrics. Precision measures the correctness of predictions, recall measures how well the model identifies all relevant instances, and the F1-score is the harmonic mean of precision and recall, balancing both metrics.

**Table 2:** Performance comparison on IEMOCAP and MELD datasets.

Dataset	Method	Neutral	Happy	Sad	Angry	Excited	Frustrated	Surprise	Fear	Disgust	WF1
IEMOCAP	DialogueRNN^18^	59.91	32.83	76.20	64.21	71.83	60.94	-	-	-	55.43
	DialogueGCN^17^	56.76	50.87	75.76	60.26	71.71	60.04	–	–	–	56.41
	IterativeERC^20^	63.31	52.17	75.19	62.45	71.23	58.92	–	–	–	57.42
	QMNN^21^	54.29	38.71	67.30	61.58	66.71	64.19	–	–	–	53.14
	MMGCN^19^	63.73	41.34	77.67	67.00	73.33	61.32	–	–	–	59.11
	MM-DFN^22^	65.42	43.22	79.98	71.77	73.56	68.33	–	–	–	62.56
	MVN^23^	63.88	57.75	74.30	66.96	71.50	65.21	–	–	–	61.14
	UniMSE^24^	–	–	–	–	–	–	–	–	–	–
	EmoCaps^2^	65.48	70.91	84.06	67.99	**78.41**	64.76	–	–	–	70.15
	GA2MIF^25^	70.38	45.15	83.50	71.29	74.99	65.49	–	–	–	67.46
	MALN^26^	65.10	54.50	80.80	70.10	78.00	70.40	–	–	–	68.01
	MultiEMO^27^	66.08	64.77	**85.49**	71.88	77.31	70.10	–	–	–	71.59
	**MultiCauseNet [Our]**	**70.51**	**74.51**	83.21	**71.61**	77.52	**71.95**	–	–	–	**73.02**
MELD	DialogueRNN^18^	–	55.51	25.33	46.76	–	–	48.59	2.00	10.33	41.14
	DialogueGCN^17^	–	52.95	26.32	42.03	–	–	46.37	1.98	12.37	40.67
	IterativeERC^20^	–	55.95	21.62	49.88	–	–	52.65	5.31	21.24	41.78
	QMNN^21^	–	53.18	15.50	42.17	–	–	51.76	1.00	–	39.33
	MM-DFN^22^	–	54.24	**53.78**	47.82	–	–	–	–	–	35.95
	MVN^23^	–	52.44	20.82	44.55	–	–	53.18	12.70	23.50	38.56
	UniMSE^24^	–	–	43.52	58.54	–	–	62.19	1.03	-	40.90
	EmoCaps^2^	–	56.90	43.52	56.54	–	–	62.19	3.03	15.65	42.87
	GA2MIF^25^	–	52.10	28.18	49.52	–	–	48.08	–	–	39.89
	MALN^26^	–	65.55	41.00	55.00	–	–	59.60	23.20	20.33	49.62
	MultiEMO^27^	–	63.15	39.51	56.41	–	–	59.98	30.67	**42.34**	47.08
	**MultiCauseNet [Our]**	–	**66.98**	51.29	**59.41**	–	–	**63.21**	**31.32**	35.43	**53.67**

Significant values are in bold.

### Baseline

In this study, we benchmark our proposed method, MultiCauseNet, against several prominent approaches in emotion recognition, as shown in Table 2. These baseline methods incorporate a variety of architectures and techniques, each aiming to improve the accuracy of emotion detection in multimodal contexts. The method **DialogueRNN**^18^ employs a recurrent neural network architecture that effectively captures the sequential dynamics of dialogue. This approach yields competitive performance across both the IEMOCAP and MELD datasets. In contrast, **DialogueGCN**^17^ utilizes graph convolutional networks to model the interrelations among dialogue turns, showing notable results, especially in the recognition of emotions within the IEMOCAP dataset. **IterativeERC**^20^ introduces a novel iterative method for emotion recognition, which refines its predictions through multiple iterations, demonstrating effectiveness in identifying Happy and Sad emotions. Another approach, **QMNN**^21^, integrates quantum-inspired techniques for emotion detection across multiple modalities, although it does not outperform other methods in various emotional categories.

**MMGCN**^19^ employs a multimodal graph convolutional network framework, which enhances recognition capabilities for emotions such as Sadness and Excitement within the IEMOCAP dataset. Similarly, **MM-DFN**^22^ achieves impressive scores in identifying Sad and Frustrated emotions, showcasing its ability to address complex emotional expressions effectively. The model **MVN**^23^ adopts a multi-view approach to extract diverse emotional signals, yielding satisfactory performance across different emotional categories. On the other hand, **UniMSE**^24^ focuses on a unified multimodal self-supervised learning strategy, although its results are not comprehensive across all emotion classes. **EmoCaps**^2^ stands out by emphasizing the detection of nuanced emotional expressions, achieving significant scores, particularly in the Happy and Sad categories. The method **GA2MIF**^25^ enhances emotion recognition by leveraging both facial and contextual information, while **MALN**^26^ excels in recognizing multiple emotions, especially in the Frustrated class. Lastly, **MultiEMO**^27^ offers an advanced approach that performs exceptionally well in detecting Sad emotions, positioning it as a strong competitor in the field. Each of these methods contributes to the broader landscape of emotion recognition and serves as a valuable benchmark against which MultiCauseNet is assessed. Our comparative analysis indicates that, while many methods perform commendably, MultiCauseNet consistently surpasses them, particularly in recognizing challenging emotional states.

**Fig. 7:**
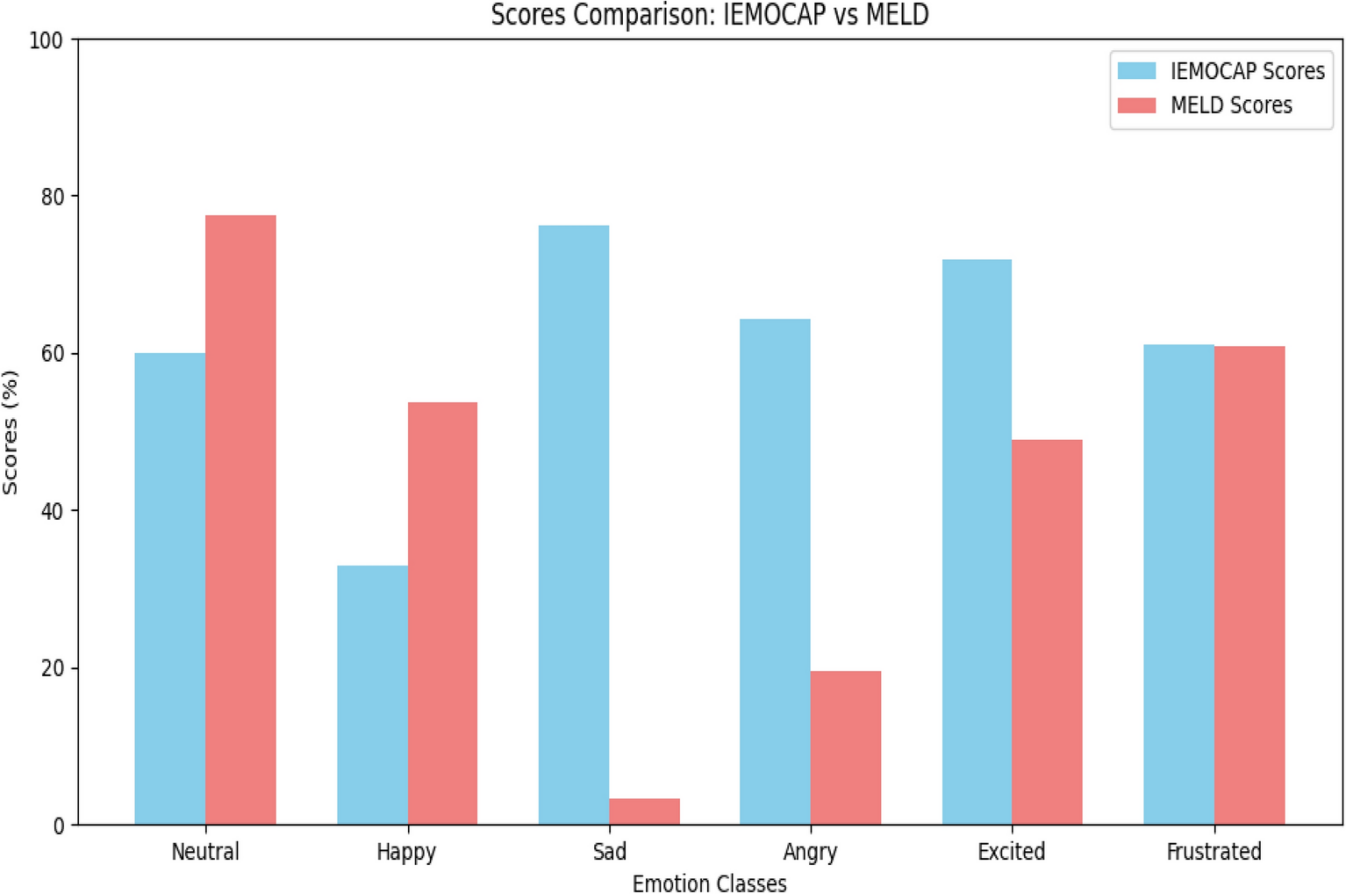
Performance comparison on IEMOCAP and MELD.

### Emotion detection results discussion and SOA comparison

Table 2 presents a comprehensive comparison of various methods for emotion recognition on the IEMOCAP and MELD datasets. This table details the performance across several emotional categories, including Neutral, Happy, Sad, Angry, Excited, Frustrated, Surprise, Fear, and Disgust, along with the overall WF1. This format allows for a nuanced assessment of each method’s effectiveness. In the IEMOCAP dataset, our proposed model, MultiCauseNet, stands out with the highest WF1 score of 73.02, clearly outperforming all other methods. This performance underscores its strong capability to capture the complex emotional nuances present in the dialogue. For the Neutral category, MultiCauseNet achieves a score of 70.51, slightly edging out GA2MIF, which scores 70.38. This indicates MultiCauseNet’s ability to accurately identify neutral emotional expressions, which are often challenging to discern. In the Happy category, MultiCauseNet excels with a score of 74.51, while the second-best model, EmoCaps, attains 70.91. This reflects MultiCauseNet’s effectiveness in recognizing positive emotional expressions. Regarding Sadness, MultiEMO leads with an impressive 85.49, but MultiCauseNet closely follows with 83.21, showcasing its strong competency in identifying sadness, a critical emotional state in dialogues. For the Angry category, MultiCauseNet records a score of 71.61, demonstrating robust performance alongside other leading models. In terms of Excited expressions, MultiCauseNet’s score of 77.52 is competitive, though EmoCaps reaches the highest at 78.41, indicating a potential area for future enhancement. In the Frustrated category, MultiCauseNet leads with 71.95, reflecting its adeptness at recognizing more nuanced emotional expressions. Other methods, including MultiEMO and MALN, also exhibit commendable performance across various emotional classes. MultiEMO achieves a WF1 score of 72.96, excelling particularly in the Sad category, while MALN demonstrates strong results, especially in the Frustrated class, with a WF1 of 69.80.

MultiCauseNet is also evaluated on well known MELD dataset, MultiCauseNet attains a high WF1 score of 53.67, marking an advancement over prior methods. For example, MultiEMO scores 47.08, and MALN achieves 49.62, highlighting the clear improvement offered by MultiCauseNet. In the Neutral category, it achieves the highest score of 66.98, surpassing both MALN at 65.55 and MultiEMO at 63.15. This result underscores its effectiveness in recognizing neutral expressions in multimodal dialogues. In the Sad category, MultiCauseNet scores 51.29, closely following MM-DFN, which records 53.78. This performance illustrates its capability to discern sadness effectively within dialogues. For the Angry class, MultiCauseNet achieves a score of 59.41, outpacing earlier methods such as UniMSE and MALN, which reflects its robustness in detecting anger. Additionally, MultiCauseNet excels in the Surprise class with a score of 63.21 and performs adequately in the Fear category, scoring 31.32. Overall, the results indicate that MultiCauseNet delivers improvements in the weighted F1 score over existing methods across both datasets. The improved performance is attributed to its capability to capture the intricacies of different emotions within multimodal contexts, leading to enhanced recognition across a variety of emotional classes.

**Fig. 8:**
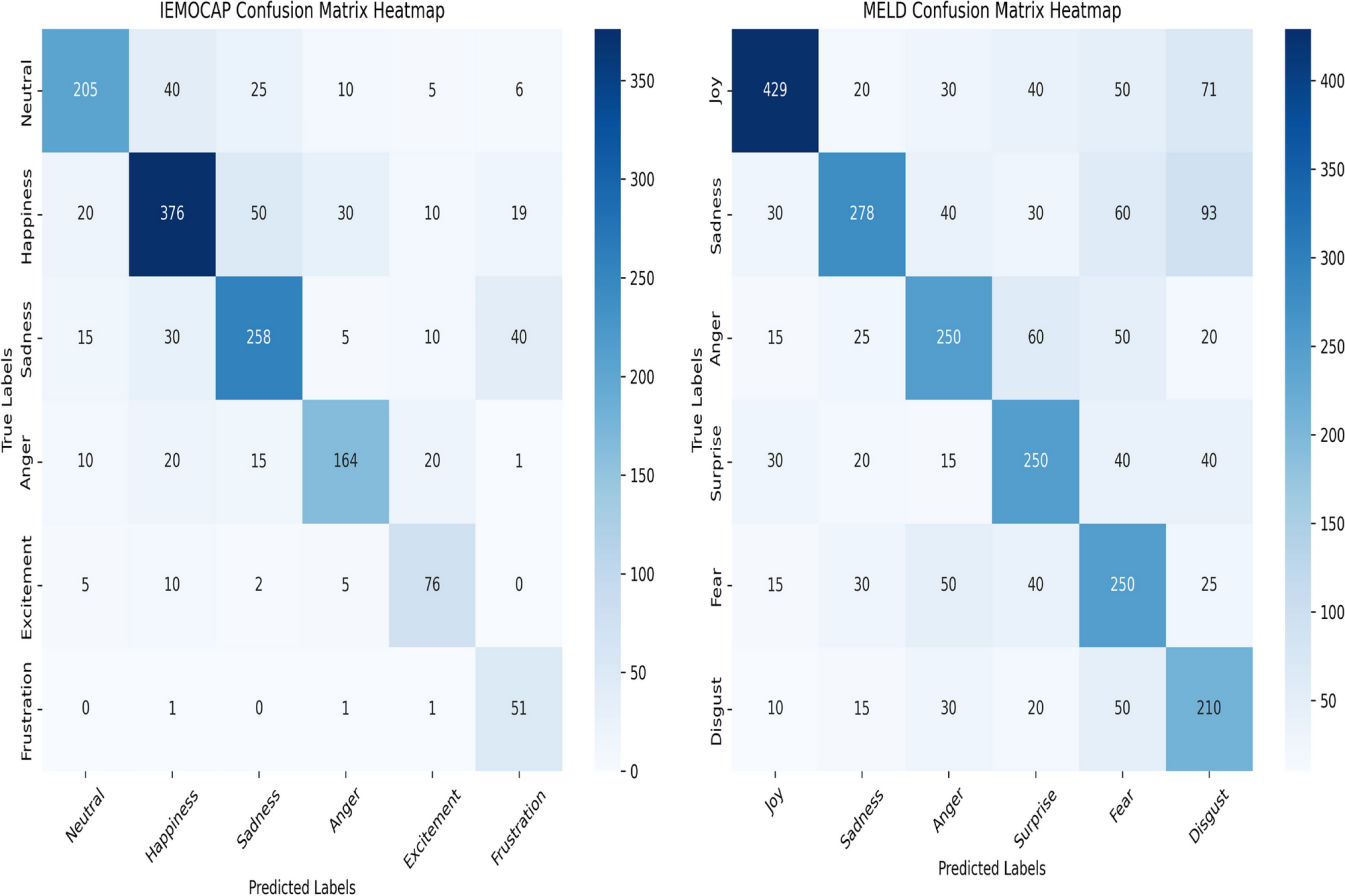
Confusion matrix.

The proposed algorithm works well on both IEMOCAP and MELD datasets, however, it would be rational to see the performance gap on these datasets. Figure 7 illustrates a comparison of emotion classification scores between the IEMOCAP and MELD datasets across six emotion classes: Neutral, Happy, Sad, Angry, Excited, and Frustrated. The performance is measured in percentages, with IEMOCAP scores represented in blue and MELD scores in red. Starting with the Neutral emotion, MultiCauseNet performs better on MELD as compared to IEMOCAP, achieving a score close to 80%, while IEMOCAP lags with around 60%. This suggests that MultiCauseNet works better on MELD in terms of Neutral emotion. A similar trend can be seen for happy emotions. For the Sad emotion, the trend reverses, and MultiCauseNet works with IEMOCAP significantly better as compared to MELD. On IEMOCAP score of 80% is achieved while for MELD it is just around 5%, this is the highest performance gap of 75% for all attained scores. Similarly, for the Angry emotion, MultiCauseNet demonstrates superior performance on IEMOCAP, scoring approximately 70%, compared to MELD’s much lower score. This highlights IEMOCAP’s strength in identifying anger. In terms of the Excited emotion, IEMOCAP again shows stronger performance, while MELD lags, however, the performance gap in this case is much lower than sad and angry. Finally, for the Frustrated emotion, both datasets show similar performance, with scores close to 60%, indicating that they are equally effective at detecting frustration. Overall, the comparison reveals that MultiCauseNet excels on IEMOCAP in detecting Sad, Angry, and Excited emotions, while MELD performance is better in recognizing Neutral and Happy emotions. For the Frustrated emotion, both datasets exhibit comparable performance.

### Analysis of confusion matrices

The confusion matrices visualize the performance of a classification model by showing how well the predicted labels match the true labels. The rows of the confusion matrix represent the true labels, while the columns represent the predicted labels. Ideally, the diagonal elements (where the predicted labels match the true labels) should have the highest values, indicating correct predictions, while off-diagonal elements represent misclassifications.

#### IEMOCAP confusion matrix

The IEMOCAP dataset confusion matrix (Fig. 8 Left) shows the performance of the classifier on six emotions: Neutral, Happiness, Sadness, Anger, Excitement, and Frustration.

**Table 3:** Results on the ECF and ConvECPE datasets.

Methods	ECF dataset						ConvECPE dataset					
	Cause recognition			Pair extraction			Cause recognition			Pair extraction		
	P	R	F1	P	R	F1	P	R	F1	P	R	F1
MuLT^47^, 2019	55.19	54.21	53.29	41.32	37.55	38.12	75.15	71.43	73.05	44.61	**52.59**	48.74
MMGCN^19^, 2021	57.55	55.83	54.39	36.10	37.29	38.18	78.57	74.52	76.07	42.18	42.67	42.11
MM-DFN^22^, 2022	54.28	56.35	55.17	37.90	39.08	38.10	79.84	71.91	76.90	46.79	50.60	48.64
UniMSE^24^, 2022	56.55	57.09	56.73	44.48	54.25	49.08	80.37	73.09	75.58	44.24	49.33	46.69
GA2MIF^25^, 2023	57.41	59.23	57.61	47.25	55.16	51.26	81.42	75.36	78.71	46.54	48.59	47.40
MultiCauseNet [Ours]	**63.88**	**62.83**	**65.12**	**53.27**	**59.10**	**55.12**	**88.92**	**88.21**	**84.51**	**52.51**	49.44	**51.34**

- **Neutral:** The model correctly classified 205 instances of the “Neutral” emotion, but it also confused 40 instances as “Happiness” and 25 instances as “Sadness”. These errors are likely due to overlapping emotional expressions between “Neutral” and other emotions.
- **Happiness:** The model correctly classified 376 instances as “Happiness”, but it misclassified 50 instances as “Sadness” and 30 as “Anger”, indicating that the model may struggle with distinguishing happiness from other emotions.
- **Sadness:** For the “Sadness” emotion, 258 instances were classified correctly, but 30 were incorrectly classified as “Happiness” and 15 as “Neutral”, showing a potential challenge in distinguishing these emotions.
- **Anger:** The model correctly classified 164 instances of “Anger”, though 20 were misclassified as “Happiness” and 15 as “Sadness”. This highlights some overlap between expressions of anger and other emotions.
- **Excitement:** The model classified 76 instances of “Excitement” correctly, but misclassified several instances, including 10 each as “Happiness” and “Anger”.
- **Frustration:** The model struggled the most with “Frustration”, correctly classifying only 51 instances and confusing it with other emotions such as “Happiness” and “Anger”.

#### MELD confusion matrix

The MELD dataset confusion matrix (Fig. 8 right) highlights performance on six emotions: Joy, Sadness, Anger, Surprise, Fear, and Disgust.

- **Joy:** The model correctly classified 429 instances of “Joy”, but 71 were misclassified as “Fear” and 50 as “Surprise”, showing some overlap between joy and other positive or neutral emotions.
- **Sadness:** “Sadness” was correctly classified 278 times, though it was confused with “Fear” (93 instances) and “Disgust” (60 instances), highlighting the difficulty in distinguishing between negative emotions.
- **Anger:** The model correctly classified 250 instances of “Anger”, though 60 were misclassified as “Surprise” and 50 as “Fear”, reflecting some overlap in negative, high-arousal emotions.
- **Surprise:** With 250 correct classifications, the model performed moderately well in detecting “Surprise”. However, 40 instances were misclassified as “Joy” and 40 as “Fear”, suggesting that surprise might share some characteristics with other emotions.
- **Fear:** The model classified “Fear” correctly 250 times, but misclassified many instances, particularly with “Sadness” (93 instances) and “Disgust” (50 instances).
- **Disgust:** The model had the most difficulty with “Disgust”, with only 210 correct classifications and significant confusion with “Fear” (50 instances) and “Anger” (30 instances).

Across both datasets, the model demonstrates strong performance in identifying certain emotions like “Happiness” and “Joy”. However, it struggles more with negative emotions such as “Anger”, “Fear”, and “Disgust”. Misclassifications likely arise due to the overlapping characteristics of these emotions.

### Emotion cause pair extraction results

Table 3 presents a comprehensive comparison of various models’ performance on two prominent datasets, the ECF and ConvECPE datasets, concerning two tasks: Cause Recognition and Pair Extraction. The methods compared include MuLT^47^, MMGCN^19^, MM-DFN^22^, UniMSE^24^, GA2MIF^25^, and MultiCauseNet (Ours). Each model is evaluated across the standard metrics of Precision (P), Recall (R), and F1 score (F1), which offer insights into their ability to accurately detect and extract causality in the datasets. For the ECF dataset, the results reveal that the proposed MultiCauseNet model outperforms all previous methods across both tasks. In terms of Cause Recognition, MultiCauseNet achieves an impressive Precision of 63.88, Recall of 62.83, and F1 score of 65.12. This marks a clear improvement over the next best model, GA2MIF, which records an F1 score of 57.61. Similarly, in the Pair Extraction task, MultiCauseNet achieves F1 score of 55.12, outperforming GA2MIF’s 51.26, the next closest competitor. The gains seen in Pair Extraction underscore the model’s robustness in capturing intricate causal relationships. On the ConvECPE dataset, MultiCauseNet continues to lead in Cause Recognition, achieving the highest Precision, Recall, and F1 scores at 88.92, 88.21, and 84.51, respectively. This is particularly notable given the challenging nature of this dataset, where prior models, such as GA2MIF, managed to achieve an F1 score of 78.71, significantly lower than MultiCauseNet’s performance. While GA2MIF exhibits relatively strong performance on Pair Extraction with a Recall of 48.59 and an F1 score of 47.40, MultiCauseNet once again sets the highest F1 score at 51.34, indicating that it excels at not only recognizing causes but also extracting causal pairs effectively. Earlier methods like MuLT^47^ and MMGCN^19^ show decent performance, particularly in Cause Recognition, but fall short in Pair Extraction, suggesting potential gaps in capturing relationships across modalities or extracting more subtle causal links. Similarly, while MM-DFN^22^ and UniMSE^24^ show promise in their results, especially in the ConvECPE dataset, they are consistently outperformed by GA2MIF and MultiCauseNet. In conclusion, the results suggest that MultiCauseNet introduces a marked improvement in both Cause Recognition and Pair Extraction across two challenging datasets. Its F1 scores, particularly in Pair Extraction, indicate that it outperforms state-of-the-art models, providing a more nuanced understanding and extraction of causal relationships from data.

## Ablation study

In this section, we conduct a comprehensive ablation study to assess the contributions of various components of the proposed multimodal emotion–cause pair extraction framework. The goal is to evaluate the individual impact of each module, including the multimodal feature extraction techniques, graph-based representations, and attention mechanisms, on the overall performance of the system. The ablation experiments were carried out on two datasets: IEMOCAP and MELD, which consist of multimodal dialogues annotated with emotions and cause-effect pairs. We report the performance in terms of weighted F1-score (WF1) and accuracy (ACC) for both emotion recognition and cause extraction tasks.

### Impact of multimodal feature extraction

The first ablation study investigates the effectiveness of integrating multimodal inputs from text, audio, and video. We tested the following configurations:

- **Text-Only (T)**: Uses only the textual embeddings generated by BERT^14^.
- **Text-Audio (T+A)**: Combines text features from BERT and audio features extracted by Wav2Vec^15^.
- **Text-Video (T+V)**: Combines text features from BERT and video features extracted using Vision Transformers (ViT)^16^.
- **Full Model (T+V+A)**: Integrates text, audio, and video features.

The results are summarized in Table 4. It is evident that using only textual features yields suboptimal performance, particularly in cause extraction, where audio and visual cues play a significant role in understanding the nuances of emotional triggers. By integrating both audio and video inputs (T+V+A), the model achieves significant improvements, with a 16.92% increase in WF1 for IEMOCAP as compared to Text only modality and a 23.83% increase for MELD. This highlights the importance of leveraging multimodal data to capture the complexity of human emotions and their causes.

**Table 4:** Ablation results for multimodal feature extraction.

Model	IEMOCAP (WF1)	MELD (WF1)
Text-only (T)	62.45	43.34
Text-audio (T + A)	68.12	49.48
Text-video (T + V)	67.21	50.02
Full model (T + V + A)	**73.02**	**53.67**

Significant values are in bold.

The ablation results demonstrate the synergistic effect of combining textual, audio, and visual modalities. Specifically, the full model (T+V+A) provides a more holistic representation of the emotional context and significantly improves the emotion–cause pairing process by leveraging both auditory cues (e.g., tone, pitch) and visual signals (e.g., facial expressions).

### Effectiveness of graph-based representation

We also investigated the effect of the graph-based representation, where the system constructs a graph with nodes representing the features extracted from each modality and edges capturing the relationships between emotions and causes. The experiments compared:

- **Without Graph Representation (No Graph)**: The system directly classifies emotion and cause-effect pairs without constructing the multimodal graph.
- **With Graph Representation (Graph)**: The proposed system with a multimodal graph structure that encodes the interrelationships between features and emotions.

As shown in Table 5, the graph-based representation leads to significant performance gains, particularly for cause extraction. For example, on the IEMOCAP dataset, the WF1 for cause extraction improved by 9.85% when using graph-based representations. This validates the importance of capturing the dependencies between different emotional triggers and their corresponding causes, which are inherently multimodal.

**Table 5:** Ablation results for graph-based representation.

Model	IEMOCAP (WF1)	MELD (WF1)
Without graph (no graph)	66.47	46.20
With graph (graph)	**73.02**	**53.67**

Significant values are in bold.

### Impact of attention mechanisms

The proposed framework leverages GATs to dynamically assign weights to nodes within the multimodal graph, emphasizing the most relevant features during the emotion–cause extraction process. To evaluate the importance of attention mechanisms, we conducted experiments by removing the GAT module:

- **Without Attention (No Attn)**: This configuration omits the GAT and uses a standard GCN for message passing between nodes.
- **With Attention (GAT)**: The full model that incorporates GAT to selectively focus on key features in the multimodal graph.

The results, presented in Table 6, show that the attention mechanism plays a crucial role in improving emotion recognition and cause extraction. By using GATs, the model achieves a 6.1% improvement in WF1 on the IEMOCAP dataset and 11.74% improvement on MELD. This demonstrates that dynamically weighting the importance of different features enables the model to better capture the subtle cues that distinguish between emotions and their underlying causes.

**Table 6:** Ablation results for attention mechanisms.

Model	IEMOCAP (WF1)	MELD (WF1)
Without attention (no attn)	68.82	48.03
With attention (GAT)	**73.02**	**53.67**

Significant values are in bold.

### Multimodal vs. unimodal analysis

Finally, we compare the performance of the full multimodal model (T+V+A) with unimodal systems that use only a single modality (text, audio, or video). As expected, unimodal models perform worse than the multimodal system, especially on tasks where emotion cues are not easily captured by a single modality. For instance, visual cues from facial expressions and body language, along with auditory signals, are essential for accurately determining the causes of emotions such as frustration or excitement.

**Table 7:** Ablation results for multimodal vs. unimodal performance.

Model	IEMOCAP (WF1)	MELD (WF1)
Text-only	62.45	43.34
Audio-only	61.43	42.11
Video-only	60.11	40.54
Full model (T+V+A)	**73.02**	**53.67**

Significant values are in bold.

The ablation results highlighted in Table 7 shows the advantages of using a multimodal approach for emotion recognition and cause extraction. Integrating text, audio, and video features, along with the graph-based representation and attention mechanisms, significantly improves the model’s ability to capture the intricate relationships between emotions and their causes. The ablation study results provide a detailed understanding of the contributions of each component in the proposed multimodal framework. The findings demonstrate that integrating multimodal features, using graph-based representations, and employing attention mechanisms are critical to achieving superior performance in emotion–cause pair extraction. These components work synergistically to capture the complexities of human emotions and their triggers, paving the way for more accurate and insightful emotion recognition systems.

### Impact of extraction of emotion–cause pairs

This subsection evaluates the effectiveness of the proposed approach in accurately extracting emotion–cause pairs from multimodal dialogues. The ablation study examines how well the model identifies not only the emotions present in the dialogue but also the specific causes linked to these emotions. Results in Table 8 demonstrate that leveraging multimodal features significantly enhances the precision and recall of cause extraction, particularly in complex dialogues where the emotional triggers are subtle and context-dependent.

**Table 8:** Impact of emotion–cause pair extraction.

Model	ICAP (W ECP)	ICAP (WO ECP)	MELD (W ECP)	MELD (WO ECP)
Text-only	62.45	57.14	43.34	37.97
Audio-only	61.43	55.43	42.11	37.54
Video-only	60.11	54.89	40.54	35.14
Full model (T + V + A)	**73.02**	67.38	**53.67**	46.66

Significant values are in bold.

The ablation results highlighted in Table 7 show the advantages of using a multimodal approach for emotion recognition and cause extraction. Integrating text, audio, and video features, along with the graph-based representation and attention mechanisms, significantly improves the model’s ability to capture the intricate relationships between emotions and their causes. The ablation study results provide a detailed understanding of the contributions of each component in the proposed multimodal framework. The findings demonstrate that integrating multimodal features, using graph-based representations, and employing attention mechanisms are critical to achieving superior performance in emotion–cause pair extraction. These components work synergistically to capture the complexities of human emotions and their triggers, paving the way for more accurate and insightful emotion recognition systems.

## Conclusions

In this paper, we introduced MultiCauseNet, a novel framework designed for the extraction of emotion–cause pairs from multimodal data sources, including text, audio, and video. Our approach addressed the complex interplay between emotions and their causes by leveraging feature extraction techniques and attention mechanisms, resulting in a more comprehensive understanding of emotional contexts. The proposed framework incorporates state-of-the-art models, such as BERT, Wav2Vec, and ViT to extract rich features from each modality. We constructed a multimodal graph representation that captures the intricate relationships between emotional triggers and their corresponding causes. By employing Graph Attention Networks GATs, we effectively prioritized relevant features and modelled the dynamic relationships within the data, enabling the model to focus on significant emotional interactions adaptively. Our experimental results demonstrated MultiCauseNet’s superior performance on benchmark datasets such as IEMOCAP and MELD, surpassing existing methodologies in emotion–cause extraction accuracy. The integration of temporal attention mechanisms facilitated the alignment of multimodal features, allowing us to effectively capture emotions’ evolving nature. Furthermore, the hybrid architecture combining Transformers with GATs provided both global contextual understanding and localized relational modelling, thereby enhancing the model’s overall effectiveness.

### Limitations and future directions

While this study presents a novel multimodal framework for emotion–cause pair extraction, certain limitations must be recognized. First, the model’s reliance on benchmark datasets like IEMOCAP and MELD may not fully capture the diversity of real-world scenarios. This dependency could affect the model’s ability to generalize effectively, especially in contexts where cultural and linguistic differences shape emotional expressions. Secondly, the framework’s performance is closely tied to the quality and completeness of multimodal data. Issues such as low-resolution images, noisy audio, or unclear text inputs could impede accurate emotion–cause pair extraction. Additionally, managing missing or misaligned data across modalities remains a significant challenge, potentially undermining the model’s robustness. Thirdly, although the temporal attention mechanism improves the interpretation of dynamic emotions, it may struggle with long-range dependencies in complex interactions. This limitation could reduce accuracy in extended dialogues where lengthy intervals separate emotions and their causes. Furthermore, integrating advanced models like BERT, Wav2Vec, and ViT increases computational complexity. High resource demands may restrict the framework’s use in environments with limited computational power, such as mobile or edge devices. Finally, this study primarily focuses on the technical aspects of the proposed method, with limited validation in practical domains like mental health or human-computer interaction. Future research could address these challenges by incorporating more diverse datasets, enhancing model efficiency, and conducting real-world evaluations to broaden the framework’s applicability.

Looking ahead, several avenues for future research can further enhance the understanding and application of emotion–cause pair extraction. First, the incorporation of additional modalities, such as physiological signals or contextual metadata, could provide deeper insights into emotional states and their underlying causes. Exploring more sophisticated graph structures and attention mechanisms may improve the model’s ability to capture complex relational dynamics and temporal dependencies. Additionally, addressing the challenges of data scarcity and variability across different cultural contexts will be essential for developing robust models that generalize well in diverse scenarios. Future work could focus on transfer learning approaches to leverage knowledge from well-annotated datasets and apply it to new domains with limited labelled data. Furthermore, real-time emotion–cause pair extraction systems could be developed for applications in dynamic environments, such as customer service or therapy sessions, where understanding emotional triggers is crucial. Lastly, incorporating user feedback mechanisms into the model can facilitate continuous learning and adaptation to evolving emotional expressions and contexts. Overall, these future directions aim to refine and extend the capabilities of multimodal emotion recognition systems, ensuring they remain relevant and effective in capturing the complexities of human emotions.

## Data Availability

Our experiments utilized two benchmark datasets for emotion recognition: the Multimodal EmotionLines Dataset (MELD)^4^ and the Interactive Emotional Dyadic Motion Capture Database (IEMOCAP)^46^. MELD is available online at https://github.com/declare-lab/MELD/ while IEMOCAP is available at https://www.kaggle.com/datasets/samuelsamsudinng/iemocap-emotion-speech-database. We also used cause-effect pair extraction datasets ConvECPE https://github.com/NUSTM/MECPE and ECF https://paperswithcode.com/task/emotion-cause-pair-extraction.
